# Burden, clinical impact, and primary antimicrobial resistance profiles of *Helicobacter pylori* infections in Cameroon: A systematic review and meta-analysis

**DOI:** 10.1371/journal.pgph.0006486

**Published:** 2026-08-11

**Authors:** Eliane V. Assokom Okoubalimba, Judith G. L. F. Ekwe Priso, Idriss Ntatou Lemouchele, Danielle C. Kedy Koum, Martin L. Koanga Mogtomo, Loick P. Kojom Foko

**Affiliations:** 1 Center for Expertise and Research in Applied Biology (CEREBA), Douala, Cameroon; 2 Department of Biochemistry, Faculty of Sciences, The University of Douala, Douala, Cameroon; 3 Department of Clinical Sciences, Faculty of Medicine and Pharmaceutical Sciences, The University of Douala, Douala, Cameroon; Faculty of Medicine, Khon Kaen University, THAILAND

## Abstract

*Helicobacter pylori* is one of the most widespread pathogens in humans and a strong predictor of gastric cancers. This systematic review and meta-analysis (PROSPERO: CRD420261375391) aimed at determining the extent, clinical impact, and antibiotic resistance profile of *H. pylori* infections in Cameroon. Six databases were exploited to identify relevant studies published between 2000 and 2026, as per PRISMA guidelines. Pooled proportions were computed using random effects models, while sensitivity analyses were performed to identify confounding factors. A total of 46 studies were included. The pooled proportion of *H. pylori* infection was 56.7% (95%CI 51.1 – 62.1%), with higher estimates found in i) clinical settings (57.0%), ii) the regions of Centre (62.2%) and Littoral (58.5%), iii) adults (59.7%), and individuals with gastrointestinal disorders (60.1%). The burden of *H. pylori* was positively correlated with gastric disease severity, with 64.5% of infection cases seen in gastric cancer. Infections have led to adverse clinical effects (e.g., anemia). *H. pylori* isolates were highly resistant to metronidazole (87.6%, 95%CI 62.4 – 96.8%), while the lowest resistant rates were found for levofloxacin (1.1%, 95%CI 0.4 - 3.2%) and rifampicin (0.6%, 95%CI 0.1 - 2.2%). An unusually high resistance rate to amoxicillin (93.4%; 95% CI 85.9–97.0%) was observed, warranting confirmatory studies. Multidrug-resistant isolates accounted for 77.6%. Several urgent challenges, including the lack of studies in four regions, in other at-risk groups (e.g., obese), limited clinical data, and resistance status vis-à-vis other commonly prescribed ATBs (i.e., amoxicillin), have been identified. This comprehensive study provides baseline data to improve therapeutic management and resistance stewardship in Cameroon.

## Introduction

*Helicobacter pylori* is one of the most widespread bacterial pathogens worldwide. This Gram-negative microaerophilic bacterium colonizes the pyloric region of the stomach of ~50% of the human population, where it can induce gastric ailments, such as ulcers, chronic gastritis, dyspepsia, and cancers [1]. The research community has made several remarkable progresses, due to knowledge and technological advancements, in the elucidation of pathophysiological mechanisms of *H. pylori* and its link with gastric cancers [1]. The bacterium is particularly well established in humans, although the exact route of transmission remains obscure.

*Helicobacter pylori* is found on all continents, with varying epidemiological distributions and risk factors. The infection with the bacterium is modulated by a complex cocktail of interrelated host, environmental, and bacterium factors (e.g., antibiotic resistance profile, human immunodeficiency virus (HIV), age, socioeconomic drivers, familial context, hygiene and sanitation) [2]. Earlier global systematic reviews and meta-analyses (SRMA) have pinpointed high *H. pylori* prevalence rates, with the highest estimated reported in Africa (current pooled estimate 52.7%) [3,4]. *Helicobacter pylori* is a modifiable etiological factor in gastric cancers [5]. Chen and colleagues observed a decrease in gastric cancer rates among adults worldwide from 1980 to 2022, a period during which *H. pylori* infection rates also declined. However, this trend was not seen in children and adolescents [4]. The treatment of *H. pylori* is often given empirically, especially in developing countries, with relatively low cure rates (e.g., ≤ 80%) for current therapies, such as proton pump inhibitors and antibiotics (ATB) (e.g., metronidazole, levofloxacin, amoxicillin, or clarithromycin), due to rising antimicrobial resistance [6–8].

In Cameroon, a country in central Africa, *H. pylori* is also a public health concern. Recent studies outlined a high prevalence of gastrointestinal cancers, with stomach cancers accounting for 15.2% of all gastrointestinal cancers in Douala [9], one of the main towns of the country. Between 2010 and 2022, *H. pylori* infection was reported to have a pooled prevalence of 57% (range 51.9 – 62.0%) in adults, but no estimate is available for children [4]. Unfortunately, this estimate was obtained from a very limited number of studies. Also, there are no reports of the extent and patterns of *H. pylori* antibiotic resistance in the country so far. In this regard, the present SRMA was conducted to determine the prevalence, clinical impact, determinants, and drug resistance patterns of *H. pylori* infections. Also, the main challenges to successful *H. pylori* control and clinical management, along with proposed solutions, are outlined to guide national healthcare and control policies.

## Materials and methods

### Guidelines and ethics

The present paper was written and registered in accordance with the Preferred Reporting Items for Systematic Reviews and Meta-analyses (PRISMA) guidelines. The PRISMA checklist is presented in S1 Table. The review protocol is registered with the International Prospective Register of Systematic Reviews (PROSPERO: CRD420261375391) and can be accessed online at https://www.crd.york.ac.uk/PROSPERO/view/CRD420261375391. Ethical clearance was not required for the study.

### Search strategy

The search for potentially eligible studies was conducted across one electronic database (PubMed), three publisher full-text platforms (African Journals Online-AJOL, ScienceDirect, and The Wiley Online Library), one aggregator platform (Ovid), and one academic metasearch engine (Google Scholar). For Google Scholar, only the results sorted by relevance were screened to enhance reproducibility. The search (i.e., syntax and controlled vocabulary) was developed for each database. The details of the search strings are presented in S2 Table.

### Eligibility criteria

All original studies (e.g., diagnostic accuracy, cross-sectional, retrospective) conducted in Cameroon, published between 2000 and 2026, written in the national languages (i.e., French and English), were eligible for the SRMA. The studies providing data on the prevalence, clinical impact, or drug resistance patterns of *H. pylori* were included. Any study addressing any of these three aims was eligible. In contrast, papers published before 2000, focused on gastrointestinal disorders (GIDs), *in vitro* studies, ethnomedicinal studies, modeling studies, and studies with no full-texts were excluded from the analysis. Also, papers (e.g., editorials, preprints, comments, conference papers) were excluded.

### Screening strategy

Titles and abstracts of records were screened independently by three authors (EVAO, JGLFEP, INL). Full texts of *prima facie* relevant studies were retained at this stage. All records were imported into Mendeley, and duplicates were removed prior to screening. The lead authors were contacted to request a copy of the paid papers for their private use. The listed references in retained papers were scrutinized to identify more studies. Consensus and discussions with the lead author LPKF were needed to solve any disagreements between authors EVAO, JGLFEP and INL.

### Data extraction

Data of interest were extracted and entered into a standardized and pilot-tested data extraction form to ensure consistency across reviewers. Extracted data were organized into several dimensions, viz., i) study characteristics (e.g., data collection year, design), ii) patient characteristics (e.g., age range, clinical presentation such as GIDs), iii) *H. pylori* detection strategy (e.g., sample, assays), iv) detection strategy outcomes (e.g., number of *H. pylori*-infected individuals), v) ATBs tested, vi) ATB resistance assays (e.g., total number of bacterial isolates tested, interpretation guidelines), and vii) drug assay outcomes (e.g., number of resistant isolates for each ATB). Finally, for antimicrobial susceptibility testing, information was extracted on the testing method (e.g., disc diffusion, agar dilution), interpretative criteria (e.g., CLSI, EUCAST), and resistance definitions adopted by each study. Where studies did not explicitly report the interpretative standard, this was recorded as “not reported.”

### Quality assessment

The Joanna Briggs Institute (JBI) Critical Appraisal tools, each designed to evaluate various study designs (e.g., cohort, cross-sectional), were used to evaluate the methodological quality of included studies [10]. These tools comprised questions with response options of “yes,” “no,” “unclear,” or “not applicable.” In line with JBI guidance, no overall quality score was calculated. Instead, each item was evaluated individually, and study quality was interpreted based on key methodological domains. The results of the appraisal were used to inform the interpretation of findings and sensitivity analyzes.

### Data management

Data were visualized using GraphPad v8.02 for Windows. Meta-analyses were performed to compute pooled proportions of *H. pylori* infection and ATB-resistant isolates. DerSimonian-Laird approach-based random-effects models (which account for both within-study and between-study variability), with the Hartung-Knapp adjustment (to obtain more reliable confidence intervals, particularly in the presence of a limited number of studies) and double arcsine Freeman-Tukey transformation (to stabilize study-specific estimates given the proportional nature of prevalence data and the potential for variance instability when proportions approach 0 or 1) [11]. Multiple estimates from a single study were included when they represented distinct subgroups (e.g., different population characteristics) or utilized different diagnostic methods relevant to the analysis. Thus, data from a particular detection assay or population group (e.g., cases and controls) were treated as a single dataset, so that if a study reported the *H. pylori* infection proportion using two assays, it was treated as two datasets. Care was taken to ensure that these datasets were non-overlapping. To minimize the impact of potential non-independence, a random-effects model was used. Two studies/datasets and a sample size of 25 were minimally required for all meta-analyses [12]. The level of heterogeneity between each study used for meta-analyzes was appraised via I^2^ and the Cochran’s Q test. Subgroup and leave-one-out analyses were used to identify sources of heterogeneity in meta-analytic assessments. Meta-regression was performed to explore potential sources of heterogeneity in the pooled estimates of *H. pylori* prevalence and antibiotic resistance patterns across studies. A random-effects meta-regression model was applied, incorporating study-level covariates such as geographic region, year of data collection, diagnostic method, sample size, or population characteristics, where available. Model assumptions and robustness of pooled estimates were evaluated through sensitivity analyses. Regression coefficients were estimated using inverse-variance weighting, and between-study variance (τ²) was accounted for within the model. The significance of moderators was assessed using Wald-type tests, with corresponding *p*-values and confidence intervals reported. Publication bias was determined using Egger’s test for small-study effects and funnel plots. Meta-analysis-related findings were visualized using forest and funnel plots. Statistical significance was set at *p* < 0.05.

## Results

### Study selection

**Fig 1:**
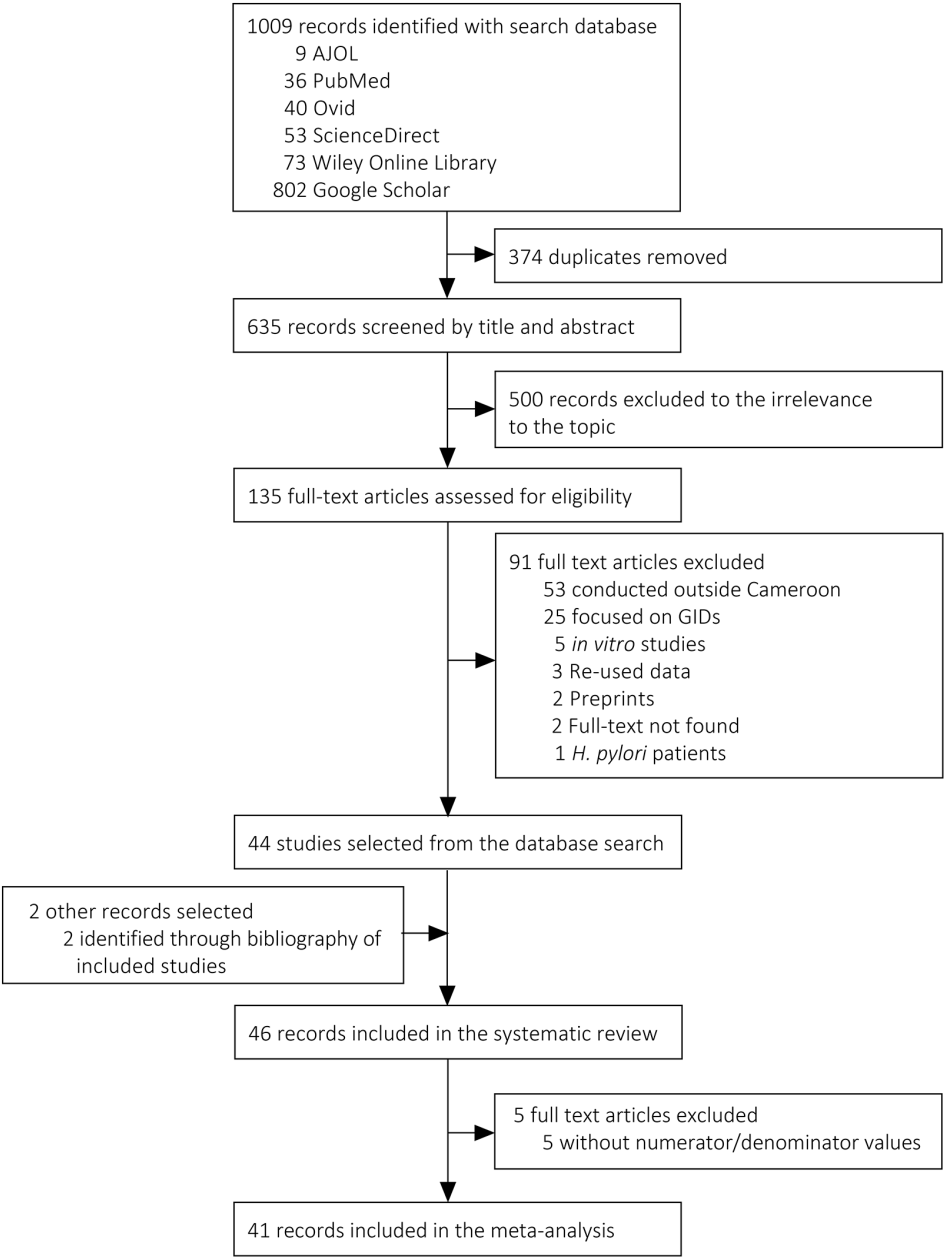
PRISMA flow diagram depicting the selection process of studies included in the systematic review and meta-analysis (A). ***Note***. AJOL: African Journals Online, GIDs: Gastrointestinal disorders. Fig 1C depicts the geographical regions of the included studies (dark blue), namely Far North (FN), North (N), Adamawa (ADA), Southwest (SW), Northwest (NW), West (W), Centre (CEN), Littoral (LT), South (S), and East (E).

**Fig 2:**
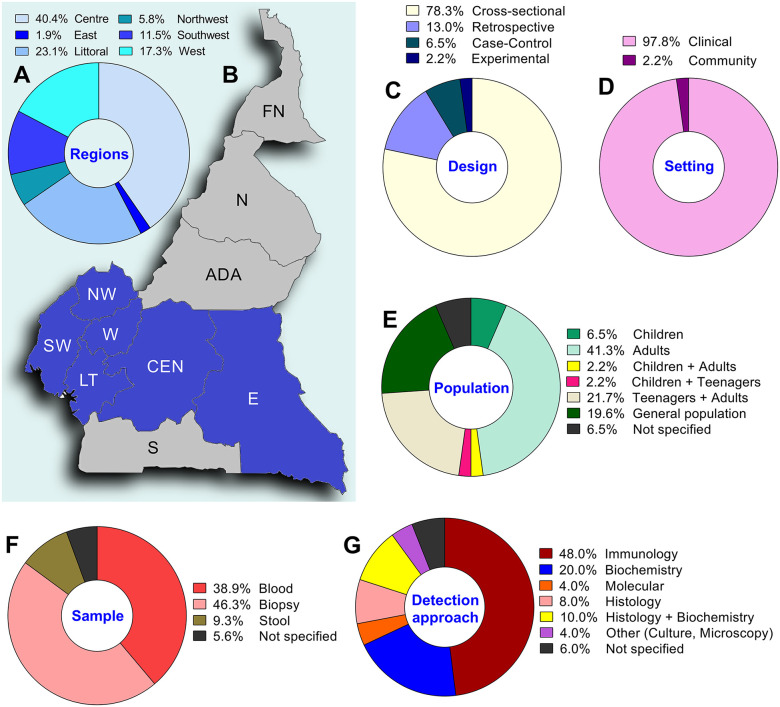
Distribution of studies with regard to regions (A, B), design (C), setting (D), populations (E), sample origin (F), and bacterial detection approaches (G). The Cameroon basemap was downloaded from Natural Earth (Base layer of the map available from: http://www.naturalearthdata.com) and visualized using the QGIS software v3.36.1 (https://qgis.org/en/site/).

A total of 46 studies were included in the systematic review. Five studies did not provide a clear numerator and/or denominator and were thus excluded from the meta-analysis. In total, 41 studies (60 epidemiology datasets and 5 drug resistance datasets) were eligible for meta-analysis, as depicted in the PRISMA flow diagram (Fig 1; S3 Table; S4 Table). The characteristics of the included studies are detailed in Fig 1 and S5 Table. The studies were mainly conducted in the regions of Centre (40.4%) and Littoral (23.1%) (Fig 2A and 2B). The bulk of studies were cross-sectional studies (78.3%), conducted in clinical settings (97.8%), and focused on adults (41.3%) (Fig 2C-2E). *Helicobacter pylori* was majorly detected in biopsies (46.3%) and blood (38.9%) (Fig 2F), using immunological-based assays targeting immunoglobulin G against bacterial antigens (Fig 2G and S6 Table). Among the included antibiotic resistance studies, susceptibility testing methods and interpretative criteria were not fully standardized. Disc diffusion (Kirby-Bauer) was the method reported in all studies. All studies explicitly referred to the 2015 CLSI recommendations [13,14], whereas one study measured MICs and used them to determine the resistance levels of *H. pylori* strains in accordance with expert recommendations [15]. Overall, the studies were well designed for six of the eight components of the JBI tools. For instance, all studies used reliable techniques for *H. pylori* detection, including a large panoply of assays. The studies had a risk of bias for two items, namely ‘detailed description of the study site’ and ‘the identification of confounding factors’. Indeed, 55.6% of studies did not address confounding factors in estimates of *H. pylori* infection (S6 Table; S1 Fig; S7 Table).

### *Helicobacter pylori* infection

**Fig 3:**
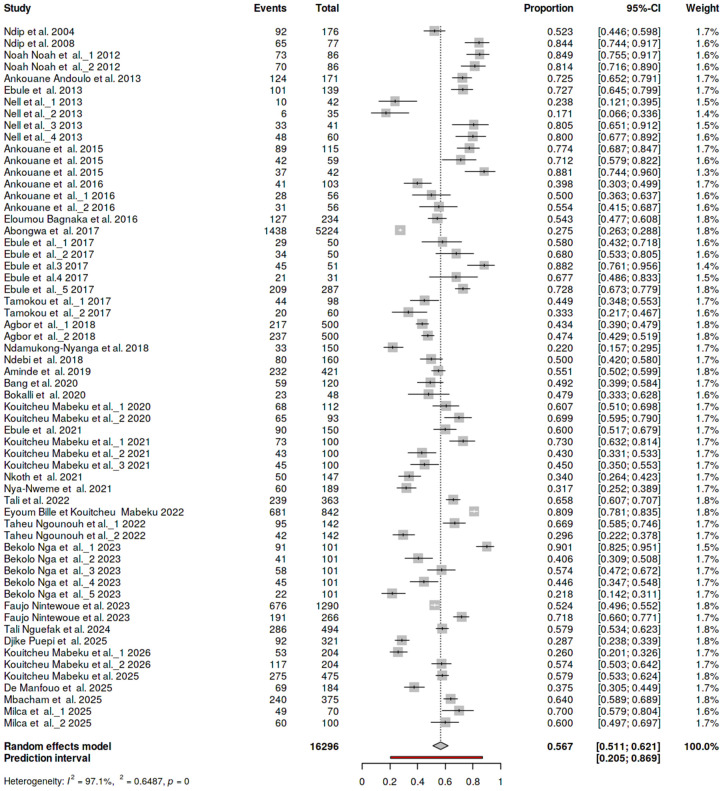
Meta-analysis of the pooled proportion of *H. pylori* infection.

The pooled proportion of *H. pylori* infection was 56.7% (95%CI 51.1 – 62.1%, I^2^ = 97.1%, *p* < 0.001) across ~17,000 individuals (Fig 3). No evidence of publication bias or impact of individual studies (leave-one-out analysis) on the pooled estimates was found (S2 Fig). Subgroup analyses revealed a significant influence of several variables on the pooled proportion estimate (Table 1; S3 Fig). The proportion of *H. pylori* infection has significantly decreased over time, from 62.8% in 2006–2010 to 49.7% after 2020. As expected, the pooled proportion was higher in clinical settings than in community settings (57.0% *vs* 50.7%). The infection was more prevalent in the regions of Centre (62.2%, 95%CI 51.6 – 71.7%, *p* < 0.0001) and Littoral (58.5%, 95%CI 46.7 – 69.4%, *p* < 0.0001), while the lowest estimate was found in the East region (45.2%, 95%CI 39.5 – 51.0%, *p* = 0.16). The burden of infection was higher in adults (59.7%, 95%CI 50.7 – 68.0%, *p* < 0.0001) than in children (37.0%, 95%CI 13.3 – 69.3%, *p* < 0.0001). Individuals with GIDs (60.1%, 95%CI 53.6 – 66.2%, *p* < 0.001) were more affected by the infection compared to their apparently healthy counterparts (45.8%, 95%CI 24.9 – 68.3%, *p* < 0.0001) (Table 1).

**Table 1:** Subgroup analysis of the pooled proportion of *H. pylori* infection.

Variables	Categories	N_S/D_	N_E_	N_P_	_p_P (%)	95%CI	I^2^ (%)	*p*
**Sampling period**	2006 - 2010	10	541	915	62.8	40.6 - 80.7	94.5	< 0.0001*
	2011 - 2015	21	4346	10306	55.8	47.4 - 63.8	98.3	< 0.0001*
	2016 - 2020	15	1693	2827	60.0	50.4 - 68.9	91.4	< 0.0001*
	2020+	14	1074	2247	49.7	36.8 - 62.8	95.2	< 0.0001*
**Study design**	Cross-sectional	48	6320	13803	54.2	47.8 - 60.6	97.5	< 0.001*
	Retrospective	6	1116	2036	60.4	41.8 - 76.4	87.9	< 0.0001*
	Case-Control	6	308	457	68.6	56.2 - 78.8	63.8	0.01*
**Setting**	Clinical	56	7557	16118	57.0	51.5 - 62.3	97.3	< 0.001*
	Community	4	97	178	50.7	7.4 - 93.0	94.3	< 0.0001*
**Regions**	Centre	19	1787	3243	62.2	51.6 - 71.7	93.5	< 0.0001*
	East	4	97	178	50.7	7.4 - 93.0	94.3	< 0.0001*
	Littoral	16	1756	2770	58.5	46.7 - 69.4	95.5	< 0.0001*
	Northwest	4	1779	5815	52.6	24.3 - 79.3	98.6	< 0.0001*
	Southwest	6	587	1262	46.3	23.6 - 70.7	97.5	< 0.0001*
	West	5	598	1318	45.2	39.5 - 51.0	37.8	0.16
	Multiple regions	6	1050	1710	61.0	52.8 - 68.7	79.0	0.0002*
**Towns**	Buea	2	93	339	26.9	1.5 - 89.8	74.7	0.04*
	Douala	16	1759	2770	58.5	46.7 - 69.4	95.5	< 0.0001*
	Dschang	3	144	318	43.9	25.2 - 64.4	58.5	0.08
	Yaoundé	19	1787	3243	62.2	51.6 - 71.7	93.5	< 0.0001*
	Multiple towns	13	2769	7609	51.6	37.7 - 65.5	98.3	< 0.0001*
**Age group**	Children	3	244	686	37.0	13.3 - 69.3	93.0	< 0.0001*
	Adults	31	1660	2904	59.7	50.7 - 68.0	92.6	< 0.0001*
	Teenagers + Adults	10	1983	3022	60.9	50.9 - 70.0	94.8	< 0.0001*
	General population	10	2710	7768	46.2	34.6 - 58.1	98.2	< 0.0001*
	Not specified	3	831	1517	65.8	20.3 - 93.6	92.6	< 0.0001*
**Clinical status**	Apparently healthy	8	419	951	45.8	24.9 - 68.3	93.9	< 0.0001*
	GIDs	40	5939	12683	60.1	53.6 - 66.2	97.7	< 0.001*
	GIDs + Other comorbidities	10	1166	2355	53.5	39.1 - 67.4	92.6	< 0.0001*
**Endoscopy**	Normal gastric mucosa	3	38	127	27.3	3.1 – 81.5	78.4	0.009*
	Inflammatory lesions	14	1622	3040	58.1	49.9 – 66.0	86.5	< 0.0001*
	Peptic ulcer diseases	5	153	268	57.0	49.1 – 64.6	0	0.48
	Premalignant lesions	2	73	136	53.8	10.1 – 92.3	7.6	0.29
	Malignant lesions	3	164	282	64.5	12.7 – 95.8	87.4	0.0003*
**Detailed endoscopy**	Esophagitis	2	70	102	68.6	40.7 – 87.4	0	0.66
	Gastritis	7	1414	2668	57.5	46.2 – 68.0	90.6	< 0.0001*
	Duodenitis	4	97	167	62.4	27.5 – 87.9	80.4	0.0016*
	Duodenal ulcer	2	34	60	56.6	6.3 – 96.2	0	0.37
	Gastric ulcer	2	96	160	60.0	26.4 – 86.2	0	0.66
	Gastric cancer	3	164	282	64.5	12.7 – 95.8	87.4	0.0003*
**Samples**	Blood	25	3635	9085	60.2	51.1 - 68.6	97.7	< 0.0001*
	Biopsy	26	3286	5495	57.1	48.6 - 65.2	94.2	< 0.0001*
	Stool	6	586	1471	38.5	26.1 - 52.6	93.4	< 0.0001*
	Not specified	3	147	245	62.4	12.1 - 95.2	91.9	< 0.0001*
**Detection principle**	Immunology	31	3799	10556	55.8	47.6 - 63.7	97.3	< 0.0001*
	Biochemistry	10	2111	3486	57.5	46.6 - 67.7	96.1	< 0.0001*
	Molecular	5	147	968	47.1	13.3 - 83.8	93.8	< 0.0001*
	Histology	4	355	573	60.1	41.0 - 76.5	82.6	0.0006*
	Histology + Biochemistry	5	614	968	63.6	48.1 - 76.7	86.3	< 0.0001*
	Other (Culture, Microscopy)	2	59	143	58.3	0.0 – 100.0	97.3	< 0.0001*
	Not specified	3	147	245	62.4	12.1 - 95.2	91.9	< 0.0001*
**Sample size**	< 100	20	773	1191	65.1	53.8 - 74.9	88.7	< 0.0001*
	100 - 499	35	3632	6749	52.9	46.4 - 59.4	94.3	< 0.0001*
	500+	5	3249	8356	51.0	25.8 - 75.6	99.5	< 0.0001*

***Note***. N_S/D_: Number of studies/datasets, N_P_: Total number of participants, N_E_: Total number of events, _p_P: Pooled proportion, 95%CI: Confidence interval at 95%, GID: Gastrointestinal disorders

The sum of dataset counts is below 60 for some variables because we did not present categories for which meta-analysis was not possible (e.g., only one dataset was available). The details are summarized in **S3 Fig**.

Statistically significant at *p < 0.05

Based on endoscopic findings, *H. pylori* infection was more frequently seen in individuals with malignant lesions (64.5%, 95%CI 12.7 – 95.8%, *p* = 0.0003) compared to those with a normal gastric mucosa (27.3%, 95%CI 3.1 – 81.5%, *p* = 0.0009). The bacterium was more frequently detected using a combination of histology and biochemistry (i.e., urease test), with a pooled proportion of 63.6% (95% CI 48.1–76.7%, *p* < 0.0001). Finally, the pooled proportion estimates were significantly lower in studies with sample sizes ≥ 500 (51%, 95%CI 25.8 – 76.6%, *p* < 0.0001) compared to those with sizes < 100 (65.1%, 95%CI 53.8 – 74.9%, *p* < 0.0001) (Table 1).

The meta-regression analysis confirmed the confounding role of data collection period, study design, age group, sample origin, and sample size (S8 Table). For instance, the odds of *H. pylori* infection were reduced by 12% (aOR = 0.88, 95% CI 0.75 – 0.98, *p* = 0.01) in studies conducted in 2020 or later, compared with 2006 – 2010. Likewise, the chances of *H. pylori* infection were reduced by 15% (aOR = 0.85, 95%CI 0.72 – 0.99, *p* = 0.042) in stool samples, by 3% (aOR = 0.97, 95%CI 0.83 – 0.93, *p* = 0.001) in studies with sample size comprised between 100 and 499, and by 9% (aOR = 0.91, 95%CI 0.81 – 0.99, *p* = 0.008) in studies with sample size ≥ 500. In contrast, the odds were increased i) by 25% in studies focused on teenagers and adults (aOR = 1.25, 95%CI 1.00 – 1.56, *p* = 0.047), compared to children, and ii) by 12% in GID patients (aOR = 1.12, 95%CI 1.02 – 1.28, *p* = 0.01) (S8 Table).

### Clinical impact of *H. pylori* infections

Very few studies addressed the clinical impact of *H. pylori* infection, with various outcomes evaluated. Thus, meta-analysis was not performed for this section, and only a narrative is presented here. Beyond the established association between *H. pylori* infection and gastrointestinal ailments (e.g., gastric cancer), as shown in the present paper, some studies addressed the role of *H. pylori* infection with extra-gastric clinical conditions (S9 Table). Overall, consistent evidence has been reported between *H. pylori* infection, total cholesterol, low-density lipoprotein cholesterol (LDL-c), and anemia. Indeed, studies consistently found that *H. pylori* infection is associated with statistically significant increased blood levels of total cholesterol, LDL-c, and proportions of anemia (S9 Table).

### Antibiotic resistance profile

Data were available for nine ATBs (i.e., amoxicillin, metronidazole, erythromycin, clarithromycin, tetracycline, ciprofloxacin, doxycycline, levofloxacin, and rifampicin) (Table 2). The analysis indicates that the highest resistance rates are observed for amoxicillin (93.4%, 95%CI 85.9 – 97.0%, *p* = 0.001), metronidazole (87.6%, 95%CI 62.4 – 96.8%, *p* < 0.0001), and erythromycin (40.1%, 95%CI 8.4 – 83.1%, *p* < 0.0001). In contrast, the lowest resistance rates were found against levofloxacin (1.1%, 95%CI 0.4 – 3.2%, *p* = 0.73) and rifampicin (0.6%, 95%CI 0.1 – 2.2%, *p* = 0.86). After excluding the study that did not use CLSI guidelines to interpret the drug-sensitivity assay, which was also the oldest one among the relevant drug susceptibility studies, the pooled resistance rates were 95.2% (95% CI 91.3–98.1%, *p* = 0.4257) for amoxicillin, 84.4% (95% CI 57.2–93.9%, *p* < 0.0001) for metronidazole, and 41.6% (95% CI 6.8–82.3%, *p* < 0.0001) for erythromycin. The pooled MDR isolates were found at a proportion of 77.6% (95%CI 49.7 – 92.4%) (Table 2).

**Table 2:** Pooled proportions of *H. pylori* resistance profile.

Parameters	N_S/D_	N_E_	N_I_	_p_P (%)	95%CI	I^2^ (%)	*p*
**Antibiotics**							
Amoxicillin	5	510	550	93.4	85.9 - 97.0	69.9	0.01*
Metronidazole	5	468	550	87.6	62.4 - 96.8	90.2	< 0.0001*
Erythromycin	4	163	418	40.1	8.4 - 83.1	94.5	< 0.0001*
Clarithromycin	5	108	550	16.6	4.2 - 47.3	94.0	< 0.0001*
Tetracycline	5	66	550	3.9	0.4 - 28.3	95.3	< 0.0001*
Ciprofloxacin	4	11	418	3.6	1.4 - 8.7	0.0	0.40
Doxycycline	4	11	418	2.9	1.0 - 8.2	17.7	0.30
Levofloxacin	4	3	418	1.1	0.4 - 3.2	0.0	0.73
Rifampicin	3	0	278	0.6	0.1 - 2.2	0.0	0.86
**Multiple antibiotic classes**							
Multidrug resistant (MDR)	4	380	505	77.6	49.7 - 92.4	90.0	< 0.0001*

***Note***. N_S/D_: Number of studies/datasets, N_I_: Total number of *H. pylori* isolates tested, N_E_: Total number of events, _p_P: Pooled proportion, 95%CI: Confidence interval at 95%

Statistically significant at *p < 0.05

## Discussion

*Helicobacter pylori* is one of the most common human pathogens. Here, using an SRMA-based approach, we have determined the proportion of *H. pylori* infections and their ATB resistance profiles in Cameroon. Key missing links, limitations, and recommendations for clinical policies are addressed (Table 3). The findings indicate a pooled *H. pylori* infection proportion of 56.7%, which is similar to pooled estimates reported in India (58%), but higher than those reported in China (42.8%) and in East Africa (50.9%) [17–19]. Such differences between estimates reflect discrepancies in *H. pylori* infection risk factors (e.g., age, lifestyle) across these regions of the globe. While the pooled prevalence offers a valuable national overview, the very high heterogeneity (I² = 97.1%) observed in this SRMA indicates considerable variability among the included studies. As a result, this estimate should be viewed as a general summary rather than an exact reflection of national prevalence. Variations in geographic locations, study populations, diagnostic methods, healthcare environments, and study timelines are likely reasons for this high heterogeneity.

**Table 3:** Challenges and solutions for *H. pylori* control in Cameroon.

Key findings, limitations, and research gaps	Recommendations
The essential part of the available *H. pylori* epidemiology and ATB resistance profile was available from six regions, especially the Centre and Littoral regions.	• It is recommended to conduct more studies in regions with limited and lacking evidence (i.e., Far North, North, Adamawa, and South).
The studies were mainly conducted in adults, mainly recruited in clinical settings. Even though the routes (e.g., oral-oral, fecal-oral) of transmission of the bacterium are not fully understood, the role of environmental drivers (e.g., poor sanitation) is crucial to its successful establishment in humans.	• Self-medication with ATBs is prevalent in the country. It is not surprising that individuals with GIDs have not attended any health facilities, but have relied on traditional medicine. Thus, community-based investigations should be conducted to determine the prevalence of *H. pylori* infection, traditional medicine practices, ATB usages, and drug resistance profile. • Given the strong role of *H. pylori* in gastric cancers, awareness campaigns should be reinforced to increase community engagement. The role of healthcare providers is crucial to achieving this objective and implementing control strategies.
Children are also at-risk groups for *H. pylori* infections. Unfortunately, only a handful of studies on the topic are available in the country.	• Basic epidemiological and clinical studies should be conducted to evaluate: • the burden and clinical impact of *H. pylori* infections in different age groups of children; • the burden of chronic and acute *H. pylori* infections; • the long-term effects of *H. pylori* infections.
Very few included studies address the determinants of *H. pylori* infections. Meta-regressions were performed, and several drivers of *H. pylori* infection were identified (i.e., age group, sample origin, and sample size), with higher odds of detection of the bacterium in teenagers, adults, and studies with large sample sizes (> 500). In contrast, the chances were reduced when using stool samples.	• Large-scale studies should be implemented in future: • to confirm the confounding role of known drivers, and analyze their patterns of association with regard to age groups (children, teenagers, adults, elderly); • to evaluate the role of increasingly incriminated factors (e.g., obesity, diabetes, concurrent infections, microbiota); • diagnostic performances of current recommended techniques as per the age groups.
The study, despite using a small number of studies, reported high resistance rates among isolates to several ATBs, especially amoxicillin, metronidazole, and erythromycin. In contrast, the most effective ATBs were levofloxacin and rifampicin. The high amoxicillin resistance rates are surprising, given that global studies indicate that the bacterium is highly sensitive to this ATB (resistance rates < 1% in most countries) [8,16]. Also, there is a dearth of clinical studies evaluating treatment failures with these ATBs used singly or in combination.	• Recommendations should cover: • Knowledge-attitudes-practice studies should be conducted in populations and health care providers to understand the resistance status; • experimental studies to confirm the current ATB resistance status for these three ATBs; • Genotyping studies should be conducted to complement the elucidation of bacterial molecular drivers of ATB resistance in the country; • ATB bioavailability studies should also be conducted; • clinical studies should be paired with *in vitro* studies to confirm the ATB resistance status; • testing additional ATBs not commonly used for *H. pylori* infections, but greatly used to treat other gastrointestinal pathogens (e.g., furazolidone, azithromycin, imipenem, minocycline) • appraising the burden and patterns of multidrug resistance.
Between-study heterogeneity was high in some meta-analyses. This is a common issue encountered when performing meta-analyses of proportions. Further detailed analyses to identify the sources of heterogeneity were not possible due to the small number of datasets for some categories (e.g., insufficient numbers for resistance profiles against imipenem or norfloxacin, subgroup analyses of the pooled proportion of *H. pylori* infections by additional comorbidities, such as diabetes).	• Future investigations, including large sample sizes, are needed; • Need to develop a standard procedure for epidemiological data on *H. pylori* infections.
Several studies found a statistically significant association between *H. pylori* infections and anemia. This is particularly interesting, given the fact that other pathogens, especially malaria parasites, can induce this condition and are highly endemic in the country.	• It would be interesting to determine the fraction of anemia cases attributable to *H. pylori* infections, and how anemic status can modulate the natural history of *H. pylori* infections in the country, especially in at-risk groups such as children.

The infection rates gradually declined over time, with the lowest estimates observed after 2020. This is likely due to improvements in standards of living and environmental conditions in Cameroon. *Helicobacter pylori* infection was less frequently seen in children than in adults, with a pooled estimate of 37%. A previous global SRMA reported an infection rate of 32.3% in children, with significant differences with regard to several factors (e.g., age, socioeconomic status, family contact, or maternal status towards *H. pylori* infection) [20]. Our findings, along with those from other reports, indicate that one-third of children worldwide carry the bacterium.

As expected, the pooled estimates of *H. pylori* infection were higher in individuals with GIDs compared to apparently healthy individuals. This finding is in line with those from previous SRMAs conducted in settings [21,22]. It is well known that this bacterium is responsible for several GIDs, although other sympatric pathogens (e.g., intestinal protozoans and helminths), also frequently detected in Cameroon, could have been responsible for GIDs. *Helicobacter pylori* bacteria induce GIDs through a complex combination of chemical, physical, and immune mechanisms (e.g., inflammation, virulence factors) [23]. Our study revealed that infection with the bacterium was more commonly observed in gastric cancer patients, with a pooled proportion of 64.5%. A similar rate (66.5%) was found in non-cardia gastric cancer cases in China [24]. *Helicobacter pylori-*associated gastritis is known to be a significant risk factor for gastric cancer [4,5]. Infection-associated inflammation in the stomach may progress to pathological changes (i.e., atrophic gastritis, metaplasia, dysplasia), which ultimately lead to malignancies (i.e., gastric cancers) [23].

The pooled estimates varied by detection approaches, with the highest rates found when combining histology (gastric biopsies) and biochemistry (urease breath test). It should be noted that there was substantial variation in the detection approaches used across studies. This variation could reflect differences in study constraints, study populations, diagnostic challenges, and funding availability. In apparently healthy individuals, it is difficult to justify invasive approaches (e.g., endoscopy and histology) over non-invasive methods using stool or blood samples. Urea breath test and stool antigen are recommended for individuals aged < 50 years and those with dyspepsia without alarm signs (e.g., weight loss, anemia, family history of gastric cancer) [25]. Histology and biochemistry can be used to test for infection (histology) and active infection (urea breath test), as well as to evaluate eradication strategies (both methods). In addition, histology provides additional information (e.g., severity of infection-associated inflammation and histomorphological features) [6]. The findings of a Cameroonian team support our statement, as serology and antigen stool were the most frequently prescribed diagnostic tests for *H. pylori* infection by primary care physicians [26].

The few clinical studies conducted in Cameroon consistently show that *H. pylori* infection increases total cholesterol and LDL-c levels. These findings are consistent with those of a recent randomized controlled trial, which found that the successful eradication of *H. pylori* infections in Slovenian patients was associated with a long-term reduction of total cholesterol, LDL-c, small dense lipoprotein, and urinary trimethylamine N-oxide [27]. These findings suggest that *H. pylori* may contribute to cardiovascular disease risk by modulating lipoprotein profiles and systemic inflammation. In addition, another group found a higher risk of dyslipidemia in individuals with persistent *H. pylori* infection [28]. These findings are particularly important given the recently reported high obesity burden in young Cameroonian adults [29], thereby outlining the need to conduct further studies on the link between *H. pylori* infection and cardiovascular diseases in the country.

*Helicobacter pylori* isolates were particularly resistant to amoxicillin (93.4%), metronidazole (87.6%), and erythromycin (40.1%), belonging to the classes of β-lactams, nitroimidazole, and macrolides, respectively. This is likely because these ATBs, in addition to proton pump inhibitors, are preferentially prescribed as first-line treatment (combined and sequential quadruple therapies) for *H. pylori* infections and other pathogens (e.g., urinary tract infections) in Cameroon [26,30,31]. Another source of increased drug pressure comes from self-medication with these drugs, which are often taken by Cameroonian populations to heal their ailments [32]. Global SRMAs have reported higher resistance rates to metronidazole (4.2 – 100%), followed by clarithromycin (12.0 – 66.7%), amoxicillin (0 – 97.1%), and levofloxacin (3.3 – 65.7%) [7,8,16,33–36]. The high level of erythromycin resistance reported here contrasts with the current global scenario (0–30%) [37]. Several factors could explain the high resistance rates of *H. pylori* isolates to these three ATBs. Self-medication with drugs, especially ATBs, is common in African populations, including Cameroonian populations [38]. Also, some Cameroonian studies reported that nearly 50% of healthcare practitioners were prescribing these ATBs without confirmatory laboratory testing [26]. This medical malpractice is an important cause of the emergence and spread of ATB-resistant bacteria, due to increased ATB pressure. It is worth noting that the high resistance rates to amoxicillin are surprising, as global studies indicate that *H. pylori* is highly sensitive to this ATB (resistance rates < 2%) [8,16]. It should also be noted that the studies used to pool resistance profiles to these ATBs included individuals with no history of recent therapy (≥2–4 weeks) (S10 Table), thereby excluding the hypothesis of retreatment patients. In this context, it would be interesting to confirm these resistance patterns in upcoming studies in the country, given the limitations of disc diffusion methods. The potential of genomic markers for tracking clarithromycin and levofloxacin resistance has been recently addressed [39]. Interestingly, MDR isolates were found at a pooled rate of 77.6%, which falls outside the range (4 – 76.4%) reported by some authors [36,40,41]. Our findings give a comprehensive insight into the current ATB resistance profile of *H. pylori* isolates, and call for rationalizing the treatment of these infections in Cameroon.

## Limitations

The findings of the present SRMA should be interpreted in light of its limitations. First, the pooled prevalence data was computed using studies conducted mostly in the Littoral, West, and Centre regions, whereas four regions of Cameroon (i.e., Far North, North, Adamawa, and South) had no published data. Consequently, the pooled estimates primarily reflect populations from a limited geographical area and should not be considered fully representative of the national population. Second, we found substantial between-study heterogeneity in meta-analyses. Furthermore, this heterogeneity is likely to arise from differences in study design, population characteristics, geographic coverage, and diagnostic method, and thus, these differences may have influenced the pooled proportion estimates and their interpretations. Consequently, the pooled prevalence should be interpreted cautiously, as it represents an average across highly diverse studies rather than an exact estimate of the national burden. Third, grey literature was excluded from the analysis. This could have helped to reduce publication bias, reduce between-study heterogeneity, and improve pooled estimates. However, we have restricted our search to peer-reviewed publications to ensure a consistent minimum standard of methodological quality and reporting, as many unpublished sources lacked sufficient detail for reliable risk-of-bias assessment and data extraction. Fourth, it was challenging to appraise the pooled estimates for the clinical impact section owing to a limited number of studies on the topic, thereby underscoring the need for further clinical studies in the country. Finally, we report high rates of resistance to amoxicillin and metronidazole, similar to those reported in studies by independent African and Asian research groups (e.g., Nigeria, China) [42,43], but higher than recent rates reported in other regions (e.g., Asia – 34%) [44]. As discussed earlier, the studies used to compute pooled estimates included samples of patients with no recent ATB therapy (2–4 weeks). Thus, the hypothesis of retreatment isolates could be excluded. These studies mainly employed the disc diffusion method and interpreted the results according to CLSI guidelines. Differences in culture conditions, zone diameter interpretation, and the lack of universally standardized breakpoints could have caused resistance misclassification. This may have contributed to the unusually high pooled estimate of amoxicillin resistance noted in this SRMA. These call for additional studies to confirm this finding (e.g., determination of the minimum inhibitory concentration of *H. pylori* isolates).

## Conclusion

The present study provides a comprehensive view of the burden and antibiotic resistance profiles of *Helicobacter pylori* infections in Cameroon. The analysis indicated a high proportion of *H. pylori* infections, with higher estimates in individuals with GIDs, teenagers and adults, blood samples, histology + biochemistry-based detection methods, and larger sample size studies. Limited evidence suggests that *H. pylori* infection may be associated with several host extra-gastric parameters (e.g., hematological and biochemical parameters), including anemia, although these observations remain preliminary. The *H. pylori* isolates are particularly resistant to three ATBs (i.e., amoxicillin, metronidazole, and erythromycin). Several urgent challenges, such as the lack of clinico-epidemiological studies in several regions of the country and resistance status vis-à-vis other commonly prescribed ATBs, especially amoxicillin, should be addressed in future. Collectively, the present findings are helpful to improve management and resistance stewardship in the country.

## Supporting information

S1 Table PRISMA checklist. (DOCX)

S2 Table Search strategies used in the review. (DOCX)

S3 Table Studies included in the systematic review and meta-analysis. (DOCX)

S4 Table Reasons for exclusion of some primarily eligible studies. (DOCX)

S5 Table Characteristics of studies included in the systematic review and meta-analysis. (DOCX)

S6 Table Detection strategies used for *H. pylori* detection. (DOCX)

S1 Fig Methodological reliability of the included studies. (DOCX)

S7 Table Individual assessment of methodological quality of the included studies. (DOCX)

S2 Fig Publication bias and leave-one-out analysis of the overall pooled proportion of *H. pylori* infection across the included studies. (DOCX)

S3 Fig Forest plots of subgroup analyses of the overall pooled proportion of *H. pylori* infection. (DOCX)

S8 Table Meta-regression analysis of potential confounders of *H. pylori* infection. (DOCX)

S9 Table Landscape of evidence on the clinical impact of *H. pylori* infections across eligible studies. (DOCX)

S10 Table Verbatim excerpts from the studies used to compute the pooled proportion of amoxicillin resistance. (DOCX)
