# Supplementary material for: Burden, clinical impact, and primary antimicrobial resistance profiles of Helicobacter pylori infections in Cameroon: A systematic review and meta-analysis
Source: PLOS Glob Public Health. 2026 Aug 11;6(8):e0006486. doi: 10.1371/journal.pgph.0006486 (PMC13460566; doi:10.1371/journal.pgph.0006486)
Supplement: Supplementary file 2 [file pgph.0006486.s002.docx]

**S2 Table**. Search strategies used in the review

| Databases | Documents | Search strategy |
| --- | --- | --- |
| PubMed | Peer-reviewed and not peer-reviewed | (“Helicobacter pylori”[MeSH Terms] OR “Helicobacter pylori” OR H. pylori) AND (“Epidemiology”[MeSH Terms] OR epidemiology OR prevalence OR incidence) AND (“Drug Resistance, Microbial”[MeSH Terms] OR antimicrobial resistance OR antibiotic resistance OR drug resistance) AND (“clinical impact” OR clinical outcomes OR disease burden) AND (“Cameroon”[MeSH Terms] OR Cameroon) AND (systematic review OR meta-analysis OR “systematic review”[Publication Type] OR “meta-analysis”[Publication Type]) AND (2000:2026[pdat]) |
| Wiley Online Library | Peer-reviewed | (“Helicobacter pylori” OR “H. pylori”) AND (epidemiology OR prevalence OR incidence) AND (“antimicrobial resistance” OR “antibiotic resistance” OR “drug resistance”) AND (“clinical impact” OR “clinical outcomes” OR morbidity) AND (Cameroon) |
| ScienceDirect | Peer-reviewed | (“Helicobacter pylori” OR “H. pylori”) AND (epidemiology OR prevalence OR incidence) AND (“antibiotic resistance” OR “antimicrobial resistance” OR “drug resistance”) AND (“clinical impact” OR “clinical outcomes”) AND (Cameroon) |
| Google scholar | Peer-reviewed and not peer-reviewed | “Helicobacter pylori” AND Cameroon AND (epidemiology OR prevalence OR incidence) AND (“antimicrobial resistance” OR “antibiotic resistance”) AND (“systematic review” OR “meta-analysis”) AND (“clinical impact” OR outcomes) |
| AJOL | Peer-reviewed | “Helicobacter pylori” AND Cameroon AND (epidemiology OR prevalence) AND (“antibiotic resistance” OR “antimicrobial resistance”) |
