# Supplementary material for: Burden, clinical impact, and primary antimicrobial resistance profiles of Helicobacter pylori infections in Cameroon: A systematic review and meta-analysis
Source: PLOS Glob Public Health. 2026 Aug 11;6(8):e0006486. doi: 10.1371/journal.pgph.0006486 (PMC13460566; doi:10.1371/journal.pgph.0006486)
Supplement: Supplementary file 3 [file pgph.0006486.s003.docx]

**S3 Table**. Studies included in the systematic review and meta-analysis

| **N** | **Studies** | **Title** |
| --- | --- | --- |
| 1 | Ndip *et al*. (2004) | *Helicobacter pylori* antigens in the faeces of asymptomatic children in the Buea and Limbe health districts of Cameroon: a pilot study |
| 2 | Ndip *et al*. (2008) | *Helicobacter pylori* isolates recovered from gastric biopsies of patients with gastro-duodenal pathologies in Cameroon: current status of antibiogram |
| 3 | Okomo Assoumou *et al*. (2012) | Association between the immune responses against *Helicobacter pylori* infections and the concentration of atrophic gastric biomarkers in Cameroon |
| 4 | Noah Noah *et al*. (2013) | Assessing GastroPanel serum markers as a non-invasive method for the diagnosis of atrophic gastritis and *Helicobacter pylori* infection |
| 5 | Ankouane Andoulo *et al*. (2013) | Epidémiologie de l’infection à *Helicobacter pylori* à Yaoundé : de la particularité à l’énigme Africaine |
| 6 | Ebule *et al*. (2013) | *Helicobacter pylori* infection and atrophic gastritis |
| 7 | Nell *et al*. (2013) | Recent acquisition of Helicobacter pylori by Baka pygmies |
| 8 | Ankouane *et al*. (2015a) | *Helicobacter pylori* infection and peptic ulcer disease in children and adolescents from the age range of 6 to 18 years old in Yaounde (Cameroon) |
| 9 | Ankouane *et al*. (2015b) | *Helicobacter pylori* and precancerous conditions of the stomach: the frequency of infection in a cross-sectional study of 79 consecutive patients with chronic antral gastritis in Yaoundé, Cameroon |
| 10 | Ankouane *et al*. (2015c) | Histological types of gastric cancer and *Helicobacter pylori* infection in Yaoundé |
| 11 | Ankouane *et al*. (2015d) | Prévalence de l'infection à *Helicobacter pylori* au travers de l'endoscopie digestive haute chez les patients VIH/sida avec des symptômes gastro-intestinaux dans les hôpitaux universitaires au Cameroun |
| 12 | Ankouane *et al*. (2016) | The prevalence of *Helicobacter pylori* infection and peptic ulcer disease in HIV-positive patients with gastrointestinal symptoms is not related to absolute CD4 Counts: A Case-Control study |
| 13 | Eloumou Bagnaka *et al*. (2016) | Facteurs de risques associés aux lésions gastroduodénales dans un hôpital de référence à Douala (Cameroun) |
| 14 | Abongwa *et al*. (2017) | Knowledge, practice and prevalence of *Helicobacter pylori* Infection in the North West Region of Cameroon |
| 15 | Ebule *et al*. (2017a) | Comparison of rapid urease and histology tests for the diagnosis of *Helicobacter pylori* infection in Cameroon |
| 16 | Ebule *et al*. (2017b) | Association of *Helicobacter pylori* infection and diabetes mellitus Type 2 subjects in Yaounde Cameroon using a panel of serum biomarkers (PGII, HpIgG): A Case Control Study |
| 17 | Ebule *et al*. (2017c) | Prevalence of *H. pylori* infection and atrophic Gastritis among dyspeptic subjects in Cameroon using a panel of serum biomarkers (PGI, PGII, G-17, HpIgG) |
| 18 | Tamokou *et al*. (2017a) | Seroprevalence of *Helicobacter pylori* IgG antibody and risk factors in hypertensive patients at Dschang District hospital in Cameroon |
| 19 | Tamokou *et al*. (2017b) | Effect of *Helicobacter pylori* infection on selected biochemical parameters of hypertensive patients at Dschang District hospital in Cameroon |
| 20 | Agbor *et al*. (2018) | Helicobacter pylori in patients with gastritis in West Cameroon: prevalence and risk factors for infection |
| 21 | Ndamukong-Nyanga *et al*. (2018) | The Influence of *Helicobacter pylori* infection on malaria parasitaemia among symptomatic patients in Buea, Cameroon |
| 22 | Ndebi *et al*. (2018) | The assessment of risk factors, lipid profile, uric acid and alanine aminotransferase in *Helicobacter pylori*-positive subjects |
| 23 | Aminde *et al*. (2019) | *Helicobacter pylori* infection among patients presenting with dyspepsia at a primary care setting in Cameroon: seroprevalence, five-year trend and predictors |
| 24 | Kouitcheu Mabeku *et al*. (2019) | Broad spectrum resistance in *Helicobacter pylori* isolated from gastric biopsies of patients with dyspepsia in Cameroon and efflux-mediated multiresistance detection in MDR isolates |
| 25 | Bang *et al*. (2020) | Clinical epidemiology and mortality risk factors of gastric cancer in a sub-Saharan African setting: a retrospective analysis of 120 cases in Yaoundé (Cameroon) |
| 26 | Bokalli *et al*. (2020) | *Helicobacter pylori* infections in peptic ulcer perforations: A retrospective analysis in two referral hospitals in Douala, Cameroon |
| 27 | Eyoum Bille *et al*. (2020) | Evolution of susceptibilities of *Helicobacter pylori* strains circulating in Cameroon to usual antibiotics: A three-year study |
| 28 | Kouitcheu Mabeku *et al*. (2020) | *Helicobacter pylori* infection, a risk factor for Type 2 diabetes mellitus: a hospital‑based cross‑sectional study among dyspeptic patients in Douala‑Cameroon |
| 29 | Ebule *et al*. (2021) | *Helicobacter pylori* infection and association with anaemia in Cameroon patients, using GastroPanel® serological biomarkers (Pepsinogen I; Pepsinogen II; Gastrin-17; Helicobacter pylori IgG) |
| 30 | Kouitcheu Mabeku *et al*. (2021) | Stool antigen testing, a reliable noninvasive method of assessment of *Helicobacter pylori* infection among patients with gastro‑duodenal disorders in Cameroon |
| 31 | Nkoth *et al*. (2021) | *Helicobacter pylori* infection and predictors risk factors among patients undergoing gastro-duodenal fibroscopy in Yaoundé, Cameroon |
| 32 | Nya-Nweme *et al*. (2021) | Association of *H. pylori* with serum iron levels and some risk factors in children aged 1-12 Years attending the Buea Regional Hospital |
| 33 | Tali *et al*. (2022) | Relationship between active *Helicobacter pylori* infection and risk factors of cardiovascular diseases, a cross-sectional hospital-based study in a Sub-Saharan setting |
| 34 | Eyoum Bille et Kouitcheu Mabeku (2022) | Relationship between active *Helicobacter pylori* infection and anemia, iron deficiency, iron deficiency anemia: A cross sectional study in a sub-Saharan setting |
| 35 | Taheu Ngounouh *et al*. (2022) | Risk factors with *Helicobacter pylori* infection prevalence among children and adult symptomatic patients attending Ad-Lucem Obobogo hospital in the health district of Efoulan, Yaoundé-Cameroon |
| 36 | Bekolo Nga *et al*. (2023) | Diagnosis of *Helicobacter pylori* infection in low out-outcome country: Rapid urease test, serological test, versus direct microbiological examination with gram staining |
| 37 | Faujo Nintewoue *et al*. (2023a) | *Helicobacter pylori* infection promotes gastric premalignancies and malignancies lesions and demotes hyperplastic polyps: A 5 Year multicentric study among Cameroonian dyspeptic patients |
| 38 | Faujo Nintewoue *et al*. (2023b) | *Helicobacter pylori* infection—A risk factor for lipid peroxidation and superoxide dismutase over-activity: A cross-sectional study among patients with dyspepsia in Cameroon |
| 39 | Tali Nguefak *et al*. (2024) | Endoscopic mucosal phenotypes and endoscopic Sydney system gastritis assessment in relation to *Helicobacter pylori* infection and upper digestive clinical signs: A 2-year study among patients with gastroduodenal disorders in Cameroon |
| 40 | Djike Puepi *et al*. (2025) | *Helicobacter pylori* antigen in stools of children: A cross-sectional study in two regional hospitals in the South West region of Cameroon |
| 41 | Kouitcheu Mabeku *et al*. (2025a) | Oxidative stress dynamic among *Helicobacter pylori*-positive patients with various stages of premalignant histologic lesions: A prospective study in Cameroon |
| 42 | Kouitcheu Mabeku *et al*. (2025b) | Rate of *Helicobacter pylori* acquisition in children and evidence of mother-child transmission in a Sub-Saharan setting |
| 43 | Kouitcheu Mabeku *et al*. (2025c) | Conventional upper-intestinal endoscopy value in predicting histopathological features related to Helicobacter pylori infection: A comparative study between endoscopy and histology findings among dyspeptic outpatients in Cameroon |
| 44 | De Manfouo *et al*. (2025) | *Helicobacter pylori* infection increases thrombocytopenia and inflammation in patients with hepatitis B and/or C: An analytical study in Cameroon |
| 45 | Mbacham *et al*. (2025) | *Helicobacter pylori* prevalence and its associated risk factors among consulting patients at the Laquintinie hospital, Douala, Cameroon |
| 46 | Milca *et al*. (2025) | Prevalence of *H. pylori* in type 2 diabetes patients attending the Nkwen District Hospital, Bamenda – Cameroon |
