# Supplementary material for: Burden, clinical impact, and primary antimicrobial resistance profiles of Helicobacter pylori infections in Cameroon: A systematic review and meta-analysis
Source: PLOS Glob Public Health. 2026 Aug 11;6(8):e0006486. doi: 10.1371/journal.pgph.0006486 (PMC13460566; doi:10.1371/journal.pgph.0006486)
Supplement: Supplementary file 4 [file pgph.0006486.s004.docx]

**S4 Table**. Reasons for exclusion of some primarily eligible studies

| **Excluded studies** | **Reasons** |
| --- | --- |
| Andoulou, F. A., *et al*. (2016). Prevalence of *Helicobacter pylori* prevalence and upper gastrointestinal endoscopy in HIV/AIDS patients with gastrointestinal symptoms in the University Teaching Hospitals in Cameroon. *Médecine et Santé Tropicales*, 26 (3), 278–282. DOI : 10.1684/mst.2016.0582 | Re-used data/Duplicate |
| Kouitcheu Mabeku, L.B., *et al*. (2018). Potential risk factors and prevalence of *Helicobacter pylori* infection among adult patients with dyspepsia symptoms in Cameroon. *BMC Infectious Diseases* 18: 278. DOI:  https://doi.org/10.1186/s12879-018-3146-1 | Re-used data/Duplicate |
| Eyoum, B. B., *et al*. (2020). Prevalence of *Helicobacter pylori* infection and relevant endoscopic features among patients with gastro-duodenal disorders, a three years cross sectional study in the Littoral Region of Cameroon. *Eur J Sci Res* 157, 247–257. | Not found (full text) |
| Laure Brigitte, N. N., Ngoe, M. N., & Ndip, L. M. (2021). Prevalence of *Helicobacter pylori* among dyspeptic patients in Buea and Limbe. *South West Health Research Bulletin* 7, 22-29. | Not found (full text) |
| Faujo Nintewoue *et al*. (2025). Prevalence of *Helicobacter pylori* and histopathological features of gastric biopsies from patients with dyspeptic symptoms in Cameroon: A contraindication of African Enigma. *International Journal of Biology* 17 (1): 2025. DOI:10.5539/ijb.v17n1p1 | Re-used data/Duplicate |
