# Supplementary material for: Burden, clinical impact, and primary antimicrobial resistance profiles of Helicobacter pylori infections in Cameroon: A systematic review and meta-analysis
Source: PLOS Glob Public Health. 2026 Aug 11;6(8):e0006486. doi: 10.1371/journal.pgph.0006486 (PMC13460566; doi:10.1371/journal.pgph.0006486)
Supplement: Supplementary file 5 [file pgph.0006486.s005.docx]

**S5 Table**. Characteristics of studies included in the systematic review and meta-analysis

| **Studies** | **Year** | **Design** | **Sites** | **Settings** | **Populations** | **Age groups** |
| --- | --- | --- | --- | --- | --- | --- |
| Ndip *et al*. | 2004 | Cross-sectional | Limbe and Buea | Clinical | Apparently healthy | Children |
| Ndip *et al*. | 2008 | Cross-sectional | Douala | Clinical | Patients with GID | not specified |
| Okomo Assoumou *et al*. | 2012 | Cross-sectional | Yaoundé | Clinical | Patients with GID | Adults |
| Noah Noah *et al*. | 2012 | Cross-sectional | Yaoundé | Clinical | Patients with GID | Adults |
| Ankouane Andoulo *et al*. | 2013 | Cross-sectional | Yaoundé | Clinical | Patients with GID | General population |
| Ebule *et al*. | 2013 | Cross-sectional | Tombel | Clinical | Patients with GID | Adults |
| Nell *et al*. | 2013 | Cross-sectional | Yokadouma and Abong Mbang | Community | Apparently healthy | Adults |
| Ankouane *et al*. | 2015 | Retrospective | Yaoundé | Clinical | Patients with GID | Children + Adolescents |
| Ankouane *et al*. | 2015 | Cross-sectional | Yaoundé | Clinical | Patients with GID | Adults |
| Ankouane *et al*. | 2015 | Retrospective | Yaoundé | Clinical | Patients with GID | Adults |
| Ankouane *et al*. | 2015 | Cross-sectional | Yaoundé | Clinical | Patients with GID | Adults |
| Ankouane *et al*. | 2016 | Cross-sectional | Douala & Yaoundé | Clinical | Patients with GID + Others HIV+ | Adults |
| Eloumou Bagnaka *et al*. | 2016 | Cross-sectional | Douala | Clinical | Patients with GID | Teenagers + Adults |
| Abongwa *et al*. | 2017 | Cross-sectional | Bamenda, Ndop, Tubah, & Kumbo | Clinical | Patients with GID | General population |
| Ebule *et al*. | 2017 | Cross-sectional | Yaoundé | Clinical | Patients with GID | Adults |
| Ebule *et al*. | 2017 | Case-Control | Yaoundé | Clinical | Diabetes mellitus | Adults |
| Ebule *et al*. | 2017 | Cross-sectional | Tombel | Clinical | Patients with GID | Adults |
| Tamokou *et al*. | 2017 | Cross-sectional | Dschang | Clinical | Hypertension | Adults |
| Tamokou *et al*. | 2017 | Cross-sectional | Dschang | Clinical | Hypertension | Adults |
| Agbor *et al*. | 2018 | Cross-sectional | Melong | Clinical | Patients with GID | General population |
| Ndamukong-Nyanga *et al*. | 2018 | Cross-sectional | Buea | Clinical | Patients with GID | General population |
| Ndebi *et al*. | 2018 | Cross-sectional | Dschang | Clinical | not specified | General population |
| Aminde *et al*. | 2019 | Retrospective | Wum | Clinical | Patients with GID | General population |
| Kouitcheu Mabeku *et al*. | 2019 | Experimental | Douala | Clinical | Patients with GID | Adults |
| Bang *et al*. | 2020 | Retrospective | Yaoundé | Clinical | Patients with GID | General population |
| Bokalli *et al*. | 2020 | Retrospective | Douala | Clinical | Patients with GID | Teenagers + Adults |
| Eyoum Bille *et al*. | 2020 | Cross-sectional | Douala | Clinical | Patients with GID | Teenagers + Adults |
| Kouitcheu Mabeku *et al*. | 2020 | Case-Control | Douala | Clinical | Patients with GID | Adults |
| Ebule *et al*. | 2021 | Cross-sectional | Yaoundé | Clinical | Anaemia | not specified |
| Kouitcheu Mabeku *et al*. | 2021 | Cross-sectional | Douala | Clinical | Patients with GID | Teenagers + Adults |
| Nkoth *et al*. | 2021 | Cross-sectional | Yaoundé | Clinical | Apparently healthy + Patients with GID | General population |
| Nya-Nweme *et al*. | 2021 | Cross-sectional | Buea | Clinical | Apparently healthy | Children |
| Tali *et al*. | 2022 | Cross-sectional | Bafoussam + Yaoundé | Clinical | Patients with GID | Teenagers + Adults |
| Eyoum Bille et Kouitcheu Mabeku | 2022 | Cross-sectional | Douala | Clinical | Patients with GID | Teenagers + Adults |
| Taheu Ngounouh *et al*. | 2022 | Cross-sectional | Yaoundé | Clinical | Patients with GID | Adults + Children |
| Bekolo Nga *et al*. | 2023 | Cross-sectional | Douala | Clinical | Patients with GID | Adults |
| Faujo Nintewoue *et al*. | 2023 | Retrospective | Yaoundé | Clinical | Patients with GID + Patients with (pre) malignancies | not specified |
| Faujo Nintewoue *et al*. | 2023 | Cross-sectional | Bafoussam + Yaoundé | Clinical | Patients with GID | Teenagers + Adults |
| Tali Nguefack *et al*. | 2024 | Cross-sectional | Bafoussam + Yaoundé | Clinical | Patients with GID | Teenagers + Adults |
| Djike Puepi *et al*. | 2025 | Cross-sectional | Limbe and Buea | Clinical | not specified | Children |
| Kouitcheu Mabeku *et al*. | 2025 | Cross-sectional | Bafoussam + Yaoundé | Clinical | Patients with GID | Teenagers + Adults |
| Kouitcheu Mabeku *et al*. | 2025 | Cross-sectional | Yaoundé | Clinical | Apparently healthy | Adults |
| Kouitcheu Mabeku *et al*. | 2025 | Cross-sectional | Bafoussam + Yaoundé | Clinical | Patients with GID | Teenagers + Adults |
| De Manfouo *et al*. | 2025 | Cross-sectional | Douala | Clinical | Hepatitis B and/or C | Adults |
| Mbacham *et al*. | 2025 | Cross-sectional | Douala | Clinical | Patients with GID | General population |
| Milca *et al*. | 2025 | Case-Control | Bamenda | Clinical | Diabetes mellitus | Adults |
