# Supplementary material for: Burden, clinical impact, and primary antimicrobial resistance profiles of Helicobacter pylori infections in Cameroon: A systematic review and meta-analysis
Source: PLOS Glob Public Health. 2026 Aug 11;6(8):e0006486. doi: 10.1371/journal.pgph.0006486 (PMC13460566; doi:10.1371/journal.pgph.0006486)
Supplement: Supplementary file 6 [file pgph.0006486.s006.docx]

**S6 Table**. Detection strategies used for *H. pylori* detection

| **Detection methods** | **Targets** | **Kits** | **Sample** | ***n*** |
| --- | --- | --- | --- | --- |
| **Biochemistry** | Labelled CO₂ produced when bacterial urease breaks down ingested labelled urea | Pronto Dry® (Medical Instruments Corporation, Solothurn, Switzerland) | Biopsy | 1 |
|  |  | Urease kit - HelicotecUT® plus (Strong Biotech Corp., Taipei 115, Taïwan) | Biopsy | 1 |
|  |  | Urease kit - (Pronto Dry®) | Biopsy | 1 |
|  |  | HelicotecUT ® Plus commercial kit (Strong Biotech Corporation, Taipei, Taiwan) | Biopsy | 2 |
|  |  | *Helicobacter pylori* AMA Rapid Urease Test 1 kit (AMAT RUT 1, Association of Medicine and Analytics, Saint Petersburg, Russia) | Biopsy | 2 |
| **ELISA** | IgG | Gastro Panel (Biohit plc Helsinki, Finland) | Blood | 1 |
|  |  | ELISA kit (Golden Bio Technologies Corp – USA) | Blood | 1 |
|  |  | HpG screen kits | Blood | 1 |
|  | IgM + IgG | System Mindray MR‐96A Elisa Chain | Blood | 1 |
| **Histology + Biochemistry** | Labelled CO₂ produced when bacterial urease breaks down ingested labelled urea | Pronto Dry® (Medical Instruments Corporation, Solothurn, Switzerland) | Biopsy | 2 |
|  |  | *Helicobacter pylori* AMA Rapid Urease Test 1 kit (AMAT RUT 1, Association of Medicine and Analytics, Saint Petersburg, Russia) | Biopsy | 2 |
| **Immunology** | Ag | *H. pylori* stool antigen (HpSA) test (Premier Platinum HpSA Meridian Diagnostics, Inc., Cincinnati, OH, USA) | Blood | 1 |
|  |  | Rapid test kit (BiOMeDica Diagnostic) | Stool | 1 |
|  |  | Immunochromatographic cassettes (WONDFO ® One Step *H. pylori* Feces Test) | Stool |  |
|  |  | Qingdao Hightop Biotech, China | Stool | 1 |
|  |  | One-step *H. pylori* antigen test device (ABON Bioparm Hangzhou, China) | Stool | 1 |
|  | IgG | not specified | Blood | 1 |
|  |  | GastroPanel (Biohit plc Helsinki, Finland) | Blood | 2 |
|  |  | One-step *H. pylori* antibody test device (DiaSpot, Indonesia) | Blood | 4 |
|  |  | *Helicobacter pylori* Antibody Test Strip | Blood | 1 |
|  |  | *Helicobacter pylori* test (Onsite Rapid Test, CTK Biotech) strip | Blood | 1 |
|  |  | *H. pylori* IgG EIA Test Kit (Rapids labs Ltds Little Bentley Colchester Essex, UK) | Blood | 1 |
|  |  | *H. pylori* One Step device | Blood | 1 |
|  | mAbs | *H. pylori* One Step Test Device (Swe-Care Rapid Diagnostic Test, for invitro diagnosis of *H. pylori* antibodies) | Blood | 1 |
|  |  | Qingdao Hightop Biotech, China | Blood | 1 |
|  |  | Monoclonal Ab kit (OEM/ODM, Zhejang Plastic, China) | Stool | 1 |
|  | IgA/IgG | *Helicobacter pylori* IgA/IgG EIA test kit, Biohit plc) | Blood | 2 |
| **Microscopy** | Whole bacterium | - | Biopsy | 1 |
| **Molecular (PCR)** | Seven housekeeping gene fragments (*atpA*, *efp*, *mutY*, *ppa*, *trpC*, *ureI*, *yphC*) | - | Biopsy | 1 |
|  | *glmM* gene | - | Biopsy | 1 |

***Note***. Ag: Antigen, ELISA: Enzyme-linked immunosorbent assay, EIA: Enzyme immunoassay, Ig: Immunoglobulin, PCR: Polymerase chain reaction, *atpA*: ATP synthase subunit alpha, *efp*: Elongation factor P, *mutY*: Adenine DNA glycosylase, *ppa*: Inorganic pyrophosphatase, *trpC*: Indole-3-glycerol phosphate synthase, *ureI*: Urease accessory protein UreI, *yphC*: GTP-binding protein (EngA), *glmM*: Phosphoglucosamine mutase (cell wall synthesis)
