# Supplementary material for: Burden, clinical impact, and primary antimicrobial resistance profiles of Helicobacter pylori infections in Cameroon: A systematic review and meta-analysis
Source: PLOS Glob Public Health. 2026 Aug 11;6(8):e0006486. doi: 10.1371/journal.pgph.0006486 (PMC13460566; doi:10.1371/journal.pgph.0006486)
Supplement: Supplementary file 8 [file pgph.0006486.s008.docx]

**S7 Table**. Individual assessment of methodological quality of the included studies

- **Cross-sectional studies**

| **Studies** | **Were the criteria for inclusion in the sample clearly defined?** | **Were the study subjects and the setting described in detail?** | **Was the exposure measured in a valid and reliable way?** | **Were objective, standard criteria used for measurement of the condition?** | **Were confounding factors identified?** | **Were strategies to deal with confounding factors stated?** | **Were the outcomes measured in a valid and reliable way?** | **Was there appropriate statistical analysis?** |
| --- | --- | --- | --- | --- | --- | --- | --- | --- |
| Ndip *et al*. (2004) | *yes* | *yes* | *yes* | *yes* | *yes* | *yes* | *yes* | *yes* |
| Ndip *et al*. (2008) | *no* | *yes* | *yes* | *yes* | *no* | *not applicable* | *yes* | *not applicable* |
| Okomo Assoumou *et al*. (2012) | *yes* | *no* | *yes* | *yes* | *no* | *not applicable* | *yes* | *yes* |
| Ankouane Andoulo *et al*. (2013) | *yes* | *yes* | *yes* | *yes* | *yes* | *no* | *yes* | *yes* |
| Nell *et al*. (2013) | *yes* | *yes* | *yes* | *yes* | *no* | *yes* | *yes* | *yes* |
| Noah Noah *et al*. (2013) | *yes* | *no* | *yes* | *yes* | *no* | *not applicable* | *yes* | *no* |
| Ebule *et al*. (2013) | *yes* | *no* | *yes* | *yes* | *yes* | *no* | *yes* | *no* |
| Ankouane *et al*. (2015b) | *yes* | *no* | *yes* | *yes* | *no* | *not applicable* | *yes* | *no* |
| Ankouane *et al*. (2015d) | *yes* | *yes* | *yes* | *yes* | *yes* | *no* | *yes* | *yes* |
| Ankouane *et al*. (2016) | *yes* | *no* | *yes* | *yes* | *yes* | *no* | *yes* | *yes* |
| Eloumou Bagnaka *et al*. (2016) | *yes* | *no* | *yes* | *yes* | *no* | *no* | *yes* | *yes* |
| Abongwa *et al*. (2017) | *yes* | *no* | *yes* | *yes* | *yes* | *no* | *yes* | *no* |
| Ebule *et al*. (2017a) | *yes* | *no* | *yes* | *yes* | *not applicable* | *no* | *yes* | *not applicable* |
| Ebule *et al*. (2017c) | *yes* | *no* | *yes* | *yes* | *yes* | *no* | *yes* | *no* |
| Tamokou *et al*. (2017a) | *yes* | *no* | *yes* | *yes* | *yes* | *no* | *yes* | *yes* |
| Tamokou *et al*. (2017b) | *yes* | *no* | *yes* | *yes* | *yes* | *no* | *yes* | *yes* |
| Agbor *et al*. (2018) | *yes* | *no* | *yes* | *yes* | *yes* | *no* | *yes* | *yes* |
| Bekolo Nga *et al*. (2018) | *yes* | *no* | *yes* | *yes* | *no* | *not applicable* | *yes* | *no* |
| Ndamukong-Nyanga *et al*. (2018) | *yes* | *no* | *yes* | *yes* | *no* | *no* | *yes* | *no* |
| Ndebi *et al*. (2018) | *yes* | *no* | *yes* | *yes* | *yes* | *yes* | *yes* | *yes* |
| Ebule *et al*. (2021) | *yes* | *no* | *yes* | *yes* | *yes* | *no* | *yes* | *no* |
| Kouitcheu Mabeku *et al*. (2021) | *yes* | *no* | *yes* | *yes* | *yes* | *no* | *yes* | *yes* |
| Nkoth *et al*. (2021) | *yes* | *no* | *yes* | *yes* | *yes* | *yes* | *yes* | *no* |
| Nya-Nweme *et al*. (2021) | *yes* | *yes* | *yes* | *yes* | *yes* | *yes* | *yes* | *yes* |
| Eyoum Bille et Kouitcheu Mabeku (2022) | *yes* | *yes* | *yes* | *yes* | *yes* | *yes* | *yes* | *yes* |
| Taheu Ngounouh *et al*. (2022) | *yes* | *no* | *yes* | *yes* | *yes* | *no* | *yes* | *yes* |
| Tali *et al*. (2022) | *yes* | *yes* | *yes* | *yes* | *yes* | *yes* | *yes* | *yes* |
| Faujo Nintewoue *et al*. (2023b) | *yes* | *yes* | *yes* | *yes* | *yes* | *yes* | *yes* | *yes* |
| Tali Nguefak *et al*. (2024) | *yes* | *yes* | *yes* | *yes* | *yes* | *no* | *yes* | *no* |
| Djike Puepi *et al*. (2025) | *yes* | *yes* | *yes* | *yes* | *yes* | *yes* | *yes* | *yes* |
| Faujo Nintewoue *et al*. (2025) | *yes* | *yes* | *yes* | *yes* | *yes* | *yes* | *yes* | *yes* |
| Kouitcheu Mabeku *et al*. (2025b) | *yes* | *yes* | *yes* | *yes* | *yes* | *yes* | *yes* | *yes* |
| Kouitcheu Mabeku *et al*. (2025c) | *yes* | *yes* | *yes* | *yes* | *yes* | *yes* | *yes* | *yes* |
| Manfouo (2025) | *yes* | *no* | *yes* | *yes* | *yes* | *yes* | *yes* | *yes* |
| Mbacham *et al*. (2025) | *yes* | *yes* | *yes* | *yes* | *yes* | *yes* | *yes* | *yes* |

- **Retrospective studies**

| **Studies** | **Were the criteria for inclusion in the sample clearly defined?** | **Were the study subjects and the setting described in detail?** | **Was the exposure measured in a valid and reliable way?** | **Were objective, standard criteria used for measurement of the condition?** | **Were confounding factors identified?** | **Were strategies to deal with confounding factors stated?** | **Were the outcomes measured in a valid and reliable way?** | **Was there appropriate statistical analysis?** |
| --- | --- | --- | --- | --- | --- | --- | --- | --- |
| Ankouane *et al*. (2015a) | *yes* | *yes* | *yes* | *yes* | *yes* | *no* | *yes* | *no* |
| Ankouane *et al*. (2015c) | *yes* | *yes* | *yes* | *yes* | *yes* | *no* | *yes* | *no* |
| Aminde *et al*. (2018) | *yes* | *yes* | *yes* | *yes* | *yes* | *yes* | *yes* | *yes* |
| Bang *et al*. (2020) | *yes* | *no* | *yes* | *yes* | *yes* | *no* | *yes* | *yes* |
| Bokalli *et al*. (2020) | *yes* | *no* | *yes* | *yes* | *yes* | *no* | *yes* | *no* |
| Faujo Nintewoue *et al*. (2023a) | *yes* | *yes* | *yes* | *yes* | *yes* | *yes* | *yes* | *yes* |

- **Case-Control studies**

| **Studies** | **Were the groups comparable  other than the presence of disease?** | **Were cases and controls  matched appropriately?** | **Were the same criteria  used for identifying  cases and controls?** | **Was exposure measured  in a standard, valid,  and reliable way?** | **Was exposure measured  the same way  for cases and controls?** | **Were confounding  factors identified?** | **Were strategies to deal  with confounding factors stated?** | **Were outcomes assessed  in a standard, valid,  and reliable way?** | **Was the exposure  period long enough  to be meaningful?** | **Was appropriate  statistical analysis  used?** |
| --- | --- | --- | --- | --- | --- | --- | --- | --- | --- | --- |
| Ebule *et al*. (2017b) | *unclear* | *unclear* | *yes* | *yes* | *yes* | *yes* | *no* | *yes* | *unclear* | *yes* |
| Kouitcheu Mabeku *et al*. (2020) | *no* | *yes* | *yes* | *yes* | *yes* | *yes* | *yes* | *yes* | *yes* | *yes* |
| Milca *et al*. (2025) | unclear | unclear | yes | yes | yes | yes | no | yes | unclear | yes |

**Not applicable for two additional studies (Experimental studies)
