# Supplementary material for: Burden, clinical impact, and primary antimicrobial resistance profiles of Helicobacter pylori infections in Cameroon: A systematic review and meta-analysis
Source: PLOS Glob Public Health. 2026 Aug 11;6(8):e0006486. doi: 10.1371/journal.pgph.0006486 (PMC13460566; doi:10.1371/journal.pgph.0006486)
Supplement: Supplementary file 9 [file pgph.0006486.s009.docx]

**S2 Fig**. Publication bias and leave-one-out analysis of the overall pooled proportion of *H. pylori* infection across the included studies

***Publication bias***

***Note***. The funnel plot indicates a potential publication bias. The Egger's test supports the presence of funnel plot asymmetry (intercept: 4.89, 95% CI: 2.64 - 7.14, t: 4.265, *p*-value = 0).

***Leave-one-out analysis***
