# Supplementary material for: Burden, clinical impact, and primary antimicrobial resistance profiles of Helicobacter pylori infections in Cameroon: A systematic review and meta-analysis
Source: PLOS Glob Public Health. 2026 Aug 11;6(8):e0006486. doi: 10.1371/journal.pgph.0006486 (PMC13460566; doi:10.1371/journal.pgph.0006486)
Supplement: Supplementary file 10 [file pgph.0006486.s010.docx]

**S3 Fig**. Forest plots of subgroup analyses of the overall pooled proportion of *H. pylori* infection

- **Sampling period**
-

- **Study design**

- **Setting**

- **Regions**

- **Towns**

- **Age group**

- **Clinical status**

- **Endoscopy**

- **Detailed endoscopy**

- **Sample**

- **Detection principle**

- **Sample size**
