# Supplementary material for: Burden, clinical impact, and primary antimicrobial resistance profiles of Helicobacter pylori infections in Cameroon: A systematic review and meta-analysis
Source: PLOS Glob Public Health. 2026 Aug 11;6(8):e0006486. doi: 10.1371/journal.pgph.0006486 (PMC13460566; doi:10.1371/journal.pgph.0006486)
Supplement: Supplementary file 11 [file pgph.0006486.s011.docx]

**S8 Table**. Meta-regression analysis of potential confounders of *H. pylori* infection

|  |  |  | **Coefficient** | | |  | **Adjusted odds ratio** | | |  |  |
| --- | --- | --- | --- | --- | --- | --- | --- | --- | --- | --- | --- |
| **Factors** | **Categories** | **N_S/D_** | **Value** | **LCL** | **UCL** |  | **Value** | **LCL** | **UCL** |  | ***p*-value** |
| **Collection period** | 2006 - 2010 | 10 | Ref. |  |  |  | Ref. |  |  |  |  |
|  | 2011 - 2015 | 21 | -0.075 | -0.211 | 0.061 |  | 0.92 | 0.81 | 1.06 |  | 0.277 |
|  | 2016 - 2020 | 15 | -0.018 | -0.166 | 0.130 |  | 0.98 | 0.84 | 1.14 |  | 0.812 |
|  | 2020+ | **14** | **-0.125** | **-0.275** | **-0.220** |  | **0.88** | **0.75** | **0.98** |  | **0.010*** |
| **Study design** | Cross-sectional | 48 | Ref. |  |  |  | Ref. |  |  |  |  |
|  | Case-Control | **6** | **0.177** | **0.010** | **0.345** |  | **1.19** | **1.01** | **1.41** |  | **0.030*** |
|  | Retrospective | 6 | 0.067 | -0.094 | 0.227 |  | 1.07 | 0.91 | 1.26 |  | 0.415 |
| **Setting** | Clinical | 56 | Ref. |  |  |  |  |  |  |  |  |
|  | Community | 4 | -0.032 | -0.225 | 0.162 |  |  |  |  |  | 0.748 |
| **Regions** | Southwest | 6 | Ref. |  |  |  | Ref. |  |  |  |  |
|  | Centre | 19 | 0.117 | -0.048 | 0.282 |  | 1.12 | 0.95 | 1.33 |  | 0.164 |
|  | East | 4 | 0.041 | -0.183 | 0.266 |  | 1.04 | 0.83 | 1.31 |  | 0.718 |
|  | Littoral | 16 | 0.082 | -0.083 | 0.247 |  | 1.09 | 0.92 | 1.28 |  | 0.329 |
|  | Northwest | 4 | -0.043 | -0.283 | 0.197 |  | 0.96 | 0.75 | 1.22 |  | 0.726 |
|  | West | 5 | -0.027 | -0.233 | 0.179 |  | 0.97 | 0.79 | 1.19 |  | 0.798 |
| **Age groups** | Children | 3 | Ref. |  |  |  | Ref. |  |  |  |  |
|  | Adults | 31 | 0.184 | -0.019 | 0.387 |  | 1.20 | 0.98 | 1.47 |  | 0.076 |
|  | Teenagers + Adults | **10** | **0.222** | **0.003** | **0.441** |  | **1.25** | **1.00** | **1.56** |  | **0.047*** |
|  | General population | 10 | 0.081 | -0.139 | 0.302 |  | 1.08 | 0.87 | 1.35 |  | 0.471 |
| **Clinical status** | Apparently healthy | 8 | Ref. |  |  |  | Ref. |  |  |  |  |
|  | GIDs | **40** | **0.113** | **0.019** | **0.248** |  | **1.12** | **1.02** | **1.28** |  | **0.01*** |
| **Sample origin** | Blood | 25 | Ref. |  |  |  | Ref. |  |  |  |  |
|  | Biopsy | 26 | 0.002 | -0.104 | 0.107 |  | 1.00 | 0.90 | 1.11 |  | 0.977 |
|  | Stool | **6** | **-0.167** | **-0.328** | **-0.006** |  | **0.85** | **0.72** | **0.99** |  | **0.042*** |
| **Detection methods** | Immunology | 31 | Ref. |  |  |  | Ref. |  |  |  |  |
|  | Biochemistry | 10 | 0.046 | -0.087 | 0.178 |  | 1.05 | 0.92 | 1.19 |  | 0.499 |
|  | Histology | 4 | 0.083 | -0.110 | 0.275 |  | 1.09 | 0.89 | 1.32 |  | 0.399 |
|  | Histology + Biochemistry | 5 | 0.100 | -0.076 | 0.276 |  | 1.11 | 0.93 | 1.32 |  | 0.266 |
|  | Molecular | 5 | -0.044 | -0.220 | 0.132 |  | 0.96 | 0.80 | 1.14 |  | 0.624 |
| **Sample size** | < 100 | 20 | Ref. |  |  |  | Ref. |  |  |  |  |
|  | 100 - 499 | **35** | **-0.026** | **-0.191** | **-0.078** |  | **0.97** | **0.83** | **0.93** |  | **0.001*** |
|  | 500+ | **5** | **-0.091** | **-0.211** | **-0.002** |  | **0.91** | **0.81** | **0.99** |  | **0.008*** |

***Note***. N_S/D_: Number of studies/datasets, Coeff.: Regression coefficient, aOR: Adjusted odds ratio, 95%CI: Confidence interval at 95%, LCL: Lower confidence limit, UCL: Upper confidence limit, Ref.: Reference group, GID: Gastro-intestinal disorders

Statistically significant at **p* < 0.05
