# Supplementary material for: Burden, clinical impact, and primary antimicrobial resistance profiles of Helicobacter pylori infections in Cameroon: A systematic review and meta-analysis
Source: PLOS Glob Public Health. 2026 Aug 11;6(8):e0006486. doi: 10.1371/journal.pgph.0006486 (PMC13460566; doi:10.1371/journal.pgph.0006486)
Supplement: Supplementary file 12 [file pgph.0006486.s012.docx]

**S9 Table**. Landscape of evidence on the clinical impact of *H. pylori* infections across eligible studies

| **Studies** | **Sites (Regions)** | **Participants** | **Findings** |
| --- | --- | --- | --- |
| Okomo Assoumou *et al*. (2012) | Yaoundé (CEN) | Adults aged ≥ 20 years with dyspepsia | - Decreased levels of pepsinogen I, but increased gastrin-17 levels, in *H. pylori*-infected patients (*p* < 0.05); - Stronger association between *H. pylori* infections, pepsinogen I, and gastrin 17 with severity of the atrophy condition (*p* < 0.05). |
| Tamokou *et al*. (2017b) | Dschang (W) | Hypertensive  adult patients | - No significant association between *H. pylori* infection and hypertension for low density lipoprotein cholesterol (LDL-c), triglycerides (TG), albumin, and potassium; - Reduced creatinine level in infected individuals in the hypertensive groups (*H. pylori*/Hypertensive vs Hypertensive only), but no significant variation in the normotensive groups (*H. pylori*/Normotensive vs Normotensive only); - Increased total cholesterol level in infected individuals in the normotensive groups (*H. pylori*/Normotensive vs Normotensive only), but no significant variation in the hypertensive groups (*H. pylori*/Hypertensive vs Hypertensive only). |
| Ndebi *et al*. (2018) | Dschang (W) | Apparently healthy adults | - Increased levels of total cholesterol, LDL-c, and uric acid in *H. pylori*-infected patients (*p* < 0.05); |
| Nya-Nweme *et al*. (2021) | Buea (SW) | Children aged 1-12 years | - Higher proportion rates of children with severe anaemia in *H. pylori*-infected patients (*p* < 0.05); |
| Eyoum Bille et Kouitcheu Mabeku (2022) | Douala (LT) | Adults aged ≥ 15 years with dyspepsia | - Higher risk of iron deficiency anaemia and low coefficient of transferrin saturation levels in *H. pylori*-infected patients (*p* < 0.05). |
| Tali *et al*. (2022) | Bafoussam (W) + Yaoundé (CEN) | Adults aged ≥ 15 years  with dyspepsia | - Increased levels of total cholesterol and LDL-c in *H. pylori*-infected patients (*p* < 0.05); - Higher risk of hyper total cholesterol and High LDL-c in *H. pylori*-infected patients (*p* < 0.05). |
| Faujo Nintewoue *et al*. (2023b) | Bafoussam (W) + Yaoundé (CEN) | Adults aged ≥ 15 years  with dyspepsia | - Increased levels of malondialdehyde and superoxide dismutase in *H. pylori*-infected patients (*p* < 0.05). |
| De Manfouo *et al*. (2025) | Douala (LT) | Adult individuals with  hepatitis B and/or C  virus infections | - Increased levels of platelets, alanine aminotransferase (ATL), aspartate aminotransferase (AST), C-reactive protein, and interleukin 6 in *H. pylori*-infected patients (*p* < 0.05); - Increased proportion of patients with high ATL and AST, and low platelet counts (thrombocytopenia) in *H. pylori*-infected patients (*p* < 0.05): |
