# Supplementary material for: Burden, clinical impact, and primary antimicrobial resistance profiles of Helicobacter pylori infections in Cameroon: A systematic review and meta-analysis
Source: PLOS Glob Public Health. 2026 Aug 11;6(8):e0006486. doi: 10.1371/journal.pgph.0006486 (PMC13460566; doi:10.1371/journal.pgph.0006486)
Supplement: Supplementary file 13 [file pgph.0006486.s013.docx]

**S10 Table**. Verbatim excerpts from the studies used to compute the pooled proportion of amoxicillin resistance

| **Studies** | **Verbatim on the inclusion criteria of the *Helicobacter pylori* samples** |
| --- | --- |
| Ndip *et al*. (2008) | ‘We enrolled only patients who gave informed consent, who had not received treatment with broad spectrum antibiotics, non-steroidal anti-inflammatory drugs or proton pump inhibitors in the previous month, and who did not have a history of dysphagia, gastric surgery or upper gastrointestinal bleeding. All patients underwent a complete physical examination and history taking by a resident gastroenterologist. Smoking and alcohol intake as well as demographic data were recorded. None of the patients had previously undergone H. pylori eradication therapy.’ |
| Kouitcheu Mabeku *et al*. (2019) | ‘Exclusion criteria were, (1) Non-cooperative patients who refused to give their consent or to participate to the study; (2) patients with a history of *H. pylori* eradication treatment; (3) patients with a history of antibiotics consumption within the last fourth weeks.’ |
| Bille Eyoum *et al*. (2020) | ‘None of the patients had received neither antimicrobial  therapy nor proton pump inhibitors for at least 2 weeks prior to endoscopy.’ |
