# Supplementary material for: Burden, clinical impact, and primary antimicrobial resistance profiles of Helicobacter pylori infections in Cameroon: A systematic review and meta-analysis
Source: PLOS Glob Public Health. 2026 Aug 11;6(8):e0006486. doi: 10.1371/journal.pgph.0006486 (PMC13460566; doi:10.1371/journal.pgph.0006486)
Supplement: Supplementary file 14 [file pgph.0006486.s007.docx]

**S1 Fig**. Methodological reliability of the included studies
