# Supplementary material for: Burden, clinical impact, and primary antimicrobial resistance profiles of Helicobacter pylori infections in Cameroon: A systematic review and meta-analysis
Source: PLOS Glob Public Health. 2026 Aug 11;6(8):e0006486. doi: 10.1371/journal.pgph.0006486 (PMC13460566; doi:10.1371/journal.pgph.0006486)
Supplement: Supplementary file 15 [file pgph.0006486.s014.docx]

**Response to Reviewers**

**Manuscript** ID: PGPH-D-26-01598

**Title**: Burden, clinical impact, and primary antimicrobial resistance profiles of *Helicobacter pylori* infections in Cameroon: a systematic review and meta-analysis

**Journal**: PLOS Global Public Health

Dear Editors-in-Chief, Dr Catherine Kyobutungi and Dr Manuela De Allegri

We thank you for the opportunity to revise and resubmit our manuscript.

In this letter, we provide a detailed, point-by-point response to all comments raised by the Editor and the Reviewers. We are grateful for the reviewers’ thoughtful and constructive feedback, which we believe has significantly improved the quality and clarity of our manuscript.

Please find below our responses to each of the reviewers’ comments (indicated in brown). All corresponding revisions have been incorporated into the revised manuscript and are highlighted in yellow for ease of reference. We confirm that all requested revisions have been carefully addressed.

We hope that the revised version meets the journal’s standards and look forward to your favorable consideration.

Sincerely,

**Loick P. Kojom Foko, PhD**

CEREBA, Douala-Cameroon

The University of Douala-Cameroon

Email: [kojomloick@gmail.com](mailto:kojomloick@gmail.com)

**Journal Requirements:**

**1 As required by our policy on Data Availability, please ensure your manuscript or supplementary information includes the following:**

**Reply**: Done.

**2 We note that your Data Availability Statement is currently as follows: "All data are within the manuscript and supporting files."**

**Reply**: This statement is correct.

**3 Some material included in your submission may be copyrighted. According to PLOS’s copyright policy, authors who use figures or other material (e.g., graphics, clipart, maps) from another author or copyright holder must demonstrate or obtain permission to publish this material under the Creative Commons Attribution 4.0 International (CC BY 4.0) License used by PLOS journals. Please closely review the details of PLOS’s copyright requirements here: PLOS Licenses and Copyright. If you need to request permissions from a copyright holder, you may use PLOS's Copyright Content Permission form.**

**Reply**: The map used for Figure 2 and the graphical abstract are not copyrighted. We have added the following point in the Figure legend:

**Fig 2**. **Distribution of studies with regard to regions (A, B), design (C), setting (D), populations (E), sample origin (F), and bacterial detection approaches (G).**

The Cameroon basemap was downloaded from Natural Earth

(Base layer of the map available from: <http://www.naturalearthdata.com>) and visualized using the QGIS software v3.36.1 (<https://qgis.org/en/site/>)

**Reviewer #1:** The manuscript is very well structured; there is a clear connection between the title, the objective, the methodology, the results, the discussion, and the conclusions. The authors also provided extensive supplementary materials that offer clarity and demonstrate the rigor applied in analyzing the selected articles.

I only have two minor recommendations for revision:

**1. The paragraph beginning on line 131 (“The review protocol is registered with the International Prospective Register of Systematic Reviews (PROSPERO: CRD420261344738) and can be accessed online at https://www.crd.york.ac.uk/PROSPERO/recorddashboard”): the link provided is not accessible, and the code shown does not correspond to this systematic literature review (SLR). Instead, it points to a different SLR ("Burden and clinico-epidemiological intricacies of genital human papillomavirus infections in Cameroonian women: a 20-year systematic review and meta-analysis 2026", link:). The correct code for this SLR is “CRD420261375391.”**

**Reply**: The reviewer is right. We have corrected this minor mistake in the abstract and the Methods section, as presented below. Thank you

The review protocol is registered with the International Prospective Register of Systematic Reviews (PROSPERO: CRD420261375391) and can be accessed online at <https://www.crd.york.ac.uk/PROSPERO/view/CRD420261375391>.

**2. Not all the sources listed as databases are, in fact, databases. Therefore, I suggest rephrasing this section as follows:**

“The search for potentially eligible studies was conducted in the following information sources: African Journals Online (AJOL), PubMed/MEDLINE, ScienceDirect, Wiley Online Library, Ovid, and Google Scholar.” Alternatively, the authors could be more specific and categorize the sources as follows: Bibliographic databases (abstract and citation databases): PubMed/MEDLINE, Publisher full-text platforms: ScienceDirect (Elsevier), Wiley Online Library, and African Journals Online (AJOL), Aggregator platform: Ovid (used to access [specify the database, e.g., MEDLINE via Ovid or Embase])., Academic metasearch engine: Google Scholar

**Reply**: We agree with you. The text has been reworded as follows:

The search for potentially eligible studies was conducted across one electronic database (PubMed), three publisher full-text platforms (African Journals Online-AJOL, ScienceDirect, and The Wiley Online Library), one aggregator platform (Ovid), and one academic metasearch engine (Google Scholar).

**Finally, I must state that I am not an expert in meta-analysis; therefore, I cannot confirm whether the statistical analysis was appropriate.**

**Reply**: We can reassure the reviewer that we have performed the statistical analyses rigorously. We have also edited some points as suggested by Reviewer 2. Thank you for this positive and constructive review.

**Reviewer #2:** Please modified some mistakes

**Major**

**1. In the Abstract (Line 51), the authors state that five databases were exploited. However, in the Materials and Methods (Line 51) and Abstract (Line 51/137), the counts conflict. The abstract says five, the methods section text says "six databases" (Line 51), but lists only five distinct platforms (AJOL, PubMed /MEDLINE, Science Direct, Wiley, Ovid) along with Google Scholar. Besides, Figure 1 shows indexing from these exact sources. Please reconcile the text to state the exact number consistently.**

**Reply**: This mistake has been solved as follows:

Six databases were exploited to identify relevant studies published between 2000 and 2026, as per PRISMA guidelines.

**2. Forest Plot vs. Abstract Discrepancy: The abstract text notes that amoxicillin resistance is 93.4% and metronidazole resistance is 87.6%. However, look at the historical data input for the first forest plot item (Figure 3): "Ndip et al. 2004" lists 02 events out of 176 totals, but reports a proportion of 0.523 (52.3%). This is mathematically impossible (1.1%). It appears to be a massive data entry error in the meta-analysis software. The authors must strictly audit all events and totals in Figure 3.**

**Reply**: Ndip *et al*. (2004) reported 92 *H. pylori* cases among 176, yielding a prevalence of 52.27%. We have checked the original paper to confirm these statistics. Figure 3 presents the pooled proportion of *H. pylori* infection, not drug resistance (Table 2). Thus, we believe no major data entry errors were introduced into the software.

**3. The "Malignant Lesions" Cohort: In Table 1, under Endoscopy, "Malignant lesions" lists 127 studies/datasets with a total participant count (N_P) of 164 and total events (N_E) of 282. It is statistically impossible to have more events than participants (282 > 164). Furthermore, the total number of studies in the review is 46, so a single subgroup cannot have 127 datasets. This row seems entirely corrupted by typographic errors or mismatched columns.**

**Reply**: We agree with the reviewer. We have corrected these typos. We have also added another supplementary file (S3 Fig) presenting the forest plots of subgroup analyses. We think that now all types have been corrected. Thank you

**4. The reported resistance rates, particularly for amoxicillin (93.4%), appear unusually high and are based on a limited number of datasets. The manuscript lacks sufficient detail regarding laboratory methods, susceptibility testing standards, and differentiation between primary and secondary resistance. These findings should be interpreted with caution and more thoroughly contextualized.**

**Reply**: We have acknowledged that this rate is unusually high. This point has been discussed in the section LIMITATIONS and Challenges (Table 3) as follows:

It is worth noting that the high resistance rates to amoxicillin are surprising, as global studies indicate that *H. pylori* is highly sensitive to this ATB (resistance rates < 2%) [8,13]. It should also be noted that the studies used to pool resistance profiles to these ATBs included individuals with no history of recent therapy (≥2-4 weeks) (**S10 Table**), thereby excluding the hypothesis of retreatment patients. In this context, it would be interesting to confirm these resistance patterns in upcoming studies in the country, given the limitations of disc diffusion methods.

**Minor**

**• Line 56-57: "The pooled proportion of H. pylori infection was 56.7% (95%CI 51.162.1%)." A hyphen or space is missing in the confidence interval range (should be 51.1–62.1%). This occurs throughout the text and tables (e.g., Lines 234, 244, 245, 247).**

**Reply**: Corrected.

**• Line 104-105: "declined in adults of several regions... but not in children and adolescents, between 1980 and 2022, where H. pylori infection prevalence has declined." The syntax here is circular and confusing. Please reword for clarity.**

**Reply**: Corrected as follows:

Chen and colleagues observed a decrease in gastric cancer rates among adults worldwide from 1980 to 2022, a period during which *H. pylori* infection rates also declined. However, this trend was not seen in children and adolescents [4].

**• Line 198: "12 and the Cochran's Q test." This is a typo for I2 (heterogeneity index). Please correct it to I2.**

**Reply**: Corrected as follows:

The level of heterogeneity between each study used for meta-analyzes was appraised via I^2^ and the Cochran’s Q test.

**• Line 265-266: "by 3 %... in studies with sample size comprised between 100 and 499". There is a stray space in "3 %", and the formatting should be tightened.**

**Reply**: Corrected.

Likewise, the chances of *H. pylori* infection were reduced by 15% (aOR = 0.85, 95%CI 0.72 – 0.99, *p* = 0.042) in stool samples, by 3% (aOR = 0.97, 95%CI 0.83 – 0.93, *p* = 0.001) in studies with sample size comprised between 100 and 499, and by 9% (aOR = 0.91, 95%CI 0.81 – 0.99, *p* = 0.008) in studies with sample size ≥ 500.

**• Line 268: Missing a period at the end of the sentence ("...sample size ≧500).**

**Reply**: Corrected as follows:

and by 9% (aOR = 0.91, 95%CI 0.81 – 0.99, *p* = 0.008) in studies with sample size ≥ 500.

**• Line 314: "The infection rates gradually declined over nearly 30 years..." However, the inclusion criteria strictly limited the search to papers published between 2000 and 2026 (a 26-year span). Modify the text to accurately reflect the timeline of your data.**

**Reply**: Corrected as follows:

The infection rates gradually declined over time, with the lowest estimates observed after 2020.

**• Table 3 formatting: The formatting of Table 3 is fragmented and messy, with recommendations awkwardly spanning cell boundaries or running across margins. It should be meticulously cleaned up to fit the journal’s stylistic requirements.**

**Reply**: This table has been revised in accordance with the Journal’s guidelines.
