# Supplementary material for: Burden, clinical impact, and primary antimicrobial resistance profiles of Helicobacter pylori infections in Cameroon: A systematic review and meta-analysis
Source: PLOS Glob Public Health. 2026 Aug 11;6(8):e0006486. doi: 10.1371/journal.pgph.0006486 (PMC13460566; doi:10.1371/journal.pgph.0006486)
Supplement: Supplementary file 16 [file pgph.0006486.s015.docx]

**Response to Reviewers**

**Manuscript** ID: PGPH-D-26-01598R1

**Title**: Burden, clinical impact, and primary antimicrobial resistance profiles of *Helicobacter pylori* infections in Cameroon: a systematic review and meta-analysis

**Journal**: PLOS Global Public Health

Dear Editors-in-Chief, Dr Catherine Kyobutungi and Dr Manuela De Allegri

We thank you for the opportunity to revise and resubmit our manuscript.

In this second letter, we provide a detailed, point-by-point response to all comments raised by the Editor and the Reviewers.

Please find below our responses to each of the reviewers’ comments (indicated in brown). All corresponding revisions have been incorporated into the revised manuscript and are highlighted in yellow for ease of reference. We confirm that all requested revisions have been carefully addressed.

We hope that the second revised version meets the journal’s standards and look forward to your favorable consideration.

Sincerely,

**Loick P. Kojom Foko, PhD**

CEREBA, Douala-Cameroon

The University of Douala-Cameroon

Email: [kojomloick@gmail.com](mailto:kojomloick@gmail.com)

**Reviewer #1: I confirm that the authors have addressed and satisfactorily completed all the revisions requested in my previous review. All comments and suggestions have been carefully considered, and the revised manuscript reflects the relevant changes. I have no further objections and recommend acceptance of the manuscript**

**Reply**: Thank you for your positive and constructive review.

**Reviewer #2: Authors had been successfully improved several critical points from the initial submission.**

**Major**

**1.Although the authors had been discuss Amoxicillin Resistance in detail, the pooled resistance rate of 93.4% remains extremely difficult to reconcile with global literature. The authors should further clarify which susceptibility testing standards were used (EUCAST, CLSI, others), whether disc diffusion methods were employed, How resistance definitions differed across studies? Whether sensitivity analyses excluding older studies or lower-quality studies change the estimate.**

**Reply**: We agree with the reviewer. We have added text detailing the standards and methods for drug susceptibility testing. We have also performed an additional meta-analysis of resistance rates to amoxicillin, excluding older studies and those without consistent definitions. Also, all relevant drug susceptibility studies had a low risk of bias. Thus, no sensitivity analysis is needed regarding study quality. We have added lines in the relevant sections of the revised paper as follows:

Methods

Finally, for antimicrobial susceptibility testing, information was extracted on the testing method (e.g., disc diffusion, agar dilution), interpretative criteria (e.g., CLSI, EUCAST), and resistance definitions adopted by each study. Where studies did not explicitly report the interpretative standard, this was recorded as "not reported".

Results

Among the included antibiotic resistance studies, susceptibility testing methods and interpretative criteria were not fully standardized. Disc diffusion (Kirby-Bauer) was the method reported in all studies. All studies explicitly referred to the 2015 CLSI recommendations [13,14], whereas one study measured MICs and used them to determine the resistance levels of *H. pylori* strains in accordance with expert recommendations [15].

After excluding the study that did not use CLSI guidelines to interpret the drug-sensitivity assay, which was also the oldest one among the relevant drug susceptibility studies, the pooled resistance rates were 95.2% (95% CI 91.3–98.1%, *p* = 0.4257) for amoxicillin, 84.4% (95% CI 57.2–93.9%, *p* < 0.0001) for metronidazole, and 41.6% (95% CI 6.8–82.3%, *p* < 0.0001) for erythromycin

Limitations

The studies used to compute pooled estimates included samples of patients with no recent ATB therapy (2-4 weeks). Thus, the hypothesis of retreatment isolates could be excluded. These studies mainly employed the disc diffusion method and interpreted the results according to CLSI guidelines. Differences in culture conditions, zone diameter interpretation, and the lack of universally standardized breakpoints could have caused resistance misclassification. This may have contributed to the unusually high pooled estimate of amoxicillin resistance noted in this SRMA. These call for additional studies to confirm this finding (e.g., determination of the minimum inhibitory concentration of *H. pylori* isolates).

**2.The pooled prevalence analysis reports: I² ≈ 97%.This level of heterogeneity is exceptionally high. Although meta-regression was conducted, much unexplained heterogeneity remains. The Discussion should better emphasize that: (1)Pooled estimates must be interpreted cautiously,(2)the summary prevalence may not represent the national burden accurately, regional and methodological differences likely contribute substantially.**

**Reply**: We have added lines in the Discussion and Limitations sections as follows:

Discussion

While the pooled prevalence offers a valuable national overview, the very high heterogeneity (I² = 97.1%) observed in this SRMA indicates considerable variability among the included studies. As a result, this estimate should be viewed as a general summary rather than an exact reflection of national prevalence. Variations in geographic locations, study populations, diagnostic methods, healthcare environments, and study timelines are likely reasons for this high heterogeneity.

Limitations

Second, we found substantial between-study heterogeneity in meta-analyses. Furthermore, this heterogeneity is likely to arise from differences in study design, population characteristics, geographic coverage, and diagnostic method, and thus, these differences may have influenced the pooled proportion estimates and their interpretations. Consequently, the pooled prevalence should be interpreted cautiously, as it represents an average across highly diverse studies rather than an exact estimate of the national burden.

**3.Four regions of Cameroon remain unrepresented. Most evidence comes from Centre and Littoral, limited National Representativeness. The authors should explicitly state that the pooled estimate primarily reflects populations from a limited geographic area rather than the entire country.**

**Reply**: We have added lines as follows:

First, the pooled prevalence data was computed using studies conducted mostly in the Littoral, West, and Centre regions, whereas four regions of Cameroon (i.e., Far North, North, Adamawa, and South) had no published data. Consequently, the pooled estimates primarily reflect populations from a limited geographical area and should not be considered fully representative of the national population.

**4.The conclusions should emphasize that these observations including H. pylori infection is associated with anemia, elevated cholesterol, elevated LDL are preliminary and hypothesis-generating rather than definitive due to evidence comes from only a few studies,**

**Reply**: We have revised the text as follows:

Limited evidence suggests that *H. pylori* infection may be associated with several host extra-gastric parameters (e.g., hematological and biochemical parameters), including anemia, although these observations remain preliminary.

**Minor**

**1. Language Editing**

**Numerous grammatical issues persist, for example:**

**"progresses" → "advances"**

**Reply**: Corrected.

**"analyzes" → "analyses"**

**Reply**: Dear Reviewer, PLOS Global Public Health recommends US English.

**Several awkward sentences throughout Introduction and Discussion.**

**Professional English editing is still recommended.**

**Reply**: Done.

**2. Reference Verification**

**Several references are very recent (2025–2026).**

**The journal should verify: accuracy,DOI validity,publication status.**

**Reply**: Done.

**3. Statistical Reporting**

**Some p-values are listed as:p = 0**

**These should be reported as:p < 0.001**

**Reply**: Corrected. These have been reported as *p* < 0.001.
