# Supplementary material for: Burden, clinical impact, and primary antimicrobial resistance profiles of Helicobacter pylori infections in Cameroon: A systematic review and meta-analysis
Source: PLOS Glob Public Health. 2026 Aug 11;6(8):e0006486. doi: 10.1371/journal.pgph.0006486 (PMC13460566; doi:10.1371/journal.pgph.0006486)
Supplement: Peer review history

# Peer review: Decision Letter 0

**DOI:** 10.1371/journal.pgph.0006486.r001

**Authors:** Kiatichai Faksri

2 Jun 2026

PGPH-D-26-01598

Burden, clinical impact, and primary antimicrobial resistance profiles of Helicobacter pylori infections in Cameroon: a systematic review and meta-analysis

PLOS Global Public Health

Dear Dr. Kojom Foko,

Thank you for submitting your manuscript to PLOS Global Public Health. After careful consideration, we feel that it has merit but does not fully meet PLOS Global Public Health’s publication criteria as it currently stands. Therefore, we invite you to submit a revised version of the manuscript that addresses the points raised during the review process.

Please submit your revised manuscript by Aug 01 2026 11:59PM. If you will need more time than this to complete your revisions, please reply to this message or contact the journal office at globalpubhealth@plos.org. When you're ready to submit your revision, log on to https://www.editorialmanager.com/pgph/ and select the 'Submissions Needing Revision' folder to locate your manuscript file.

Please include the following items when submitting your revised manuscript:

- A letter that responds to each point raised by the editor and reviewer(s). You should upload this letter as a separate file labeled 'Response to Reviewers'.
- A marked-up copy of your manuscript that highlights changes made to the original version. You should upload this as a separate file labeled 'Revised Manuscript with Track Changes'.
- An unmarked version of your revised paper without tracked changes. You should upload this as a separate file labeled 'Manuscript'.

Guidelines for resubmitting your figure files are available below the reviewer comments at the end of this letter.

As the corresponding author, your ORCID iD is verified in the submission system and will appear in the published article. PLOS supports the use of ORCID, and we encourage all coauthors to register for an ORCID iD and use it as well. Please encourage your coauthors to verify their ORCID iD within the submission system before final acceptance, as unverified ORCID iDs will not appear in the published article. *Only*  the individual author can complete the verification step; PLOS staff *cannot*  verify ORCID iDs on behalf of authors.

We look forward to receiving your revised manuscript.

Kind regards,

Kiatichai Faksri, Ph.D

Academic Editor

PLOS Global Public Health

Journal Requirements:

1. As required by our policy on Data Availability, please ensure your manuscript or supplementary information includes the following:

A numbered table of all studies identified in the literature search, including those that were excluded from the analyses.

For every excluded study, the table should list the reason(s) for exclusion.

If any of the included studies are unpublished, include a link (URL) to the primary source or detailed information about how the content can be accessed.

A table of all data extracted from the primary research sources for the systematic review and/or meta-analysis. The table must include the following information for each study:

Name of data extractors and date of data extraction

Confirmation that the study was eligible to be included in the review.

All data extracted from each study for the reported systematic review and/or meta-analysis that would be needed to replicate your analyses.

If data or supporting information were obtained from another source (e.g. correspondence with the author of the original research article), please provide the source of data and dates on which the data/information were obtained by your research group.

If applicable for your analysis, a table showing the completed risk of bias and quality/certainty assessments for each study or outcome.  Please ensure this is provided for each domain or parameter assessed. For example, if you used the Cochrane risk-of-bias tool for randomized trials, provide answers to each of the signalling questions for each study. If you used GRADE to assess certainty of evidence, provide judgements about each of the quality of evidence factor. This should be provided for each outcome.

An explanation of how missing data were handled.

This information can be included in the main text, supplementary information, or relevant data repository. Please note that providing these underlying data is a requirement for publication in this journal, and if these data are not provided your manuscript might be rejected.

2. We note that your Data Availability Statement is currently as follows: "All data are within the manuscript and supporting files."

Please confirm at this time whether or not your submission contains all raw data required to replicate the results of your study. Authors must share the “minimal data set” for their submission. PLOS defines the minimal data set to consist of the data required to replicate all study findings reported in the article, as well as related metadata and methods (https://journals.plos.org/plosone/s/data-availability#loc-minimal-data-set-definition).

For example, authors should submit the following data:

- The values behind the means, standard deviations and other measures reported;

- The values used to build graphs;

- The points extracted from images for analysis.

Authors do not need to submit their entire data set if only a portion of the data was used in the reported study.

If your submission does not contain these data, please either upload them as Supporting Information files or deposit them to a stable, public repository and provide us with the relevant URLs, DOIs, or accession numbers. For a list of recommended repositories, please see https://journals.plos.org/plosone/s/recommended-repositories.

If there are ethical or legal restrictions on sharing a de-identified data set, please explain them in detail (e.g., data contain potentially sensitive information, data are owned by a third-party organization, etc.) and who has imposed them (e.g., an ethics committee). Please also provide contact information for a data access committee, ethics committee, or other institutional body to which data requests may be sent. If data are owned by a third party, please indicate how others may request data access.

3. Some material included in your submission may be copyrighted. According to PLOS’s copyright policy, authors who use figures or other material (e.g., graphics, clipart, maps) from another author or copyright holder must demonstrate or obtain permission to publish this material under the Creative Commons Attribution 4.0 International (CC BY 4.0) License used by PLOS journals. Please closely review the details of PLOS’s copyright requirements here: PLOS Licenses and Copyright. If you need to request permissions from a copyright holder, you may use PLOS's Copyright Content Permission form.

Please respond directly to this email or email the journal office and provide any known details concerning your material's license terms and permissions required for reuse, even if you have not yet obtained copyright permissions or are unsure of your material's copyright compatibility.

Potential Copyright Issues:

a. Graphical abstract.jpg : Please confirm whether you drew the images / clip-art within the figure panels by hand. If you did not draw the images, please provide (a) a link to the source of the images or icons and their license / terms of use; or (b) written permission from the copyright holder to publish the images or icons under our CC-BY 4.0 license. Alternatively, you may replace the images with open source alternatives. See these open source resources you may use to replace images / clip-art:

- https://commons.wikimedia.org

- https://openclipart.org/

b. "Graphical abstract.jpg" and Figure 2: please (a) provide a direct link to the base layer of the map (i.e., the country or region border shape) and ensure this is also included in the figure legend; and (b) provide a link to the terms of use / license information for the base layer image or shapefile. We cannot publish proprietary or copyrighted maps (e.g. Google Maps, Mapquest) and the terms of use for your map base layer must be compatible with our CC-BY 4.0 license.

Note: if you created the map in a software program like R or ArcGIS, please locate and indicate the source of the basemap shapefile onto which data has been plotted.

If your map was obtained from a copyrighted source please amend the figure so that the base map used is from an openly available source. Alternatively, please provide explicit written permission from the copyright holder granting you the right to publish the material under our CC-BY 4.0 license.

Please note that the following CC BY licenses are compatible with PLOS license: CC BY 4.0, CC BY 2.0 and CC BY 3.0, meanwhile such licenses as CC BY-ND 3.0 and others are not compatible due to additional restrictions.

If you are unsure whether you can use a map or not, please do reach out and we will be able to help you. The following websites are good examples of where you can source open access or public domain maps:

* U.S. Geological Survey (USGS) - All maps are in the public domain. (http://www.usgs.gov)

* PlaniGlobe - All maps are published under a Creative Commons license so please cite “PlaniGlobe, http://www.planiglobe.com, CC BY 2.0” in the image credit after the caption. (http://www.planiglobe.com/?lang=enl)

* Natural Earth - All maps are public domain. (http://www.naturalearthdata.com/about/terms-of-use/)

If the reviewer comments include a recommendation to cite specific previously published works, please review and evaluate these publications to determine whether they are relevant and should be cited. There is no requirement to cite these works unless the editor has indicated otherwise.

Additional Editor Comments (if provided):

[Note: HTML markup is below. Please do not edit.]

Reviewers' comments:

Reviewer's Responses to Questions

**Comments to the Author**

1. Does this manuscript meet PLOS Global Public Health’s publication criteria?

Reviewer #1: Yes

Reviewer #2: Yes

**********

2. Has the statistical analysis been performed appropriately and rigorously?

Reviewer #1: I don't know

Reviewer #2: No

**********

3. Have the authors made all data underlying the findings in their manuscript fully available (please refer to the Data Availability Statement at the start of the manuscript PDF file)?

The PLOS Data policy

Reviewer #1: Yes

Reviewer #2: No

**********

4. Is the manuscript presented in an intelligible fashion and written in standard English?

Reviewer #1: Yes

Reviewer #2: No

**********

Reviewer #1: The manuscript is very well structured; there is a clear connection between the title, the objective, the methodology, the results, the discussion, and the conclusions. The authors also provided extensive supplementary materials that offer clarity and demonstrate the rigor applied in analyzing the selected articles.

I only have two minor recommendations for revision:

1. The paragraph beginning on line 131 (“The review protocol is registered with the International Prospective Register of Systematic Reviews (PROSPERO: CRD420261344738) and can be accessed online at https://www.crd.york.ac.uk/PROSPERO/recorddashboard”): the link provided is not accessible, and the code shown does not correspond to this systematic literature review (SLR). Instead, it points to a different SLR ("Burden and clinico-epidemiological intricacies of genital human papillomavirus infections in Cameroonian women: a 20-year systematic review and meta-analysis 2026", link:). The correct code for this SLR is “CRD420261375391.”

2. Not all the sources listed as databases are, in fact, databases. Therefore, I suggest rephrasing this section as follows:

“The search for potentially eligible studies was conducted in the following information sources: African Journals Online (AJOL), PubMed/MEDLINE, ScienceDirect, Wiley Online Library, Ovid, and Google Scholar.”

Alternatively, the authors could be more specific and categorize the sources as follows:

Bibliographic databases (abstract and citation databases): PubMed/MEDLINE

Publisher full-text platforms: ScienceDirect (Elsevier), Wiley Online Library, and African Journals Online (AJOL)

Aggregator platform: Ovid (used to access [specify the database, e.g., MEDLINE via Ovid or Embase]).

Academic metasearch engine: Google Scholar

Finally, I must state that I am not an expert in meta-analysis; therefore, I cannot confirm whether the statistical analysis was appropriate.

Reviewer #2: Please modified some mistakes

1. In the Abstract (Line 51), the authors state that five databases were exploited. However, in the Materials and Methods (Line 51) and Abstract (Line 51/137), the counts conflict. The abstract says five, the methods section text says "six databases" (Line 51), but lists only five distinct platforms (AJOL, PubMed /MEDLINE, Science Direct, Wiley, Ovid) along with Google Scholar. Besides, Figure 1 shows indexing from these exact sources. Please reconcile the text to state the exact number consistently.

2. Forest Plot vs. Abstract Discrepancy: The abstract text notes that amoxicillin resistance is 93.4% and metronidazole resistance is 87.6%. However, look at the historical data input for the first forest plot item (Figure 3): "Ndip et al. 2004" lists 02 events out of 176 totals, but reports a proportion of 0.523 (52.3%). This is mathematically impossible ( 1.1%). It appears to be a massive data entry error in the meta-analysis software. The authors must strictly audit all events and totals in Figure 3.

3. The "Malignant Lesions" Cohort: In Table 1, under Endoscopy, "Malignant lesions" lists 127 studies/datasets with a total participant count (N_P) of 164 and total events (N_E) of 282. It is statistically impossible to have more events than participants (282 > 164). Furthermore, the total number of studies in the review is 46, so a single subgroup cannot have 127 datasets. This row seems entirely corrupted by typographic errors or mismatched columns.

4. The reported resistance rates, particularly for amoxicillin (93.4%), appear unusually high and are based on a limited number of datasets. The manuscript lacks sufficient detail regarding laboratory methods, susceptibility testing standards, and differentiation between primary and secondary resistance. These findings should be interpreted with caution and more thoroughly contextualized.

Minor

• Line 56-57: "The pooled proportion of H. pylori infection was 56.7% (95%CI 51.162.1%)." A hyphen or space is missing in the confidence interval range (should be 51.1–62.1%). This occurs throughout the text and tables (e.g., Lines 234, 244, 245, 247).

• Line 104-105: "declined in adults of several regions... but not in children and adolescents, between 1980 and 2022, where H. pylori infection prevalence has declined." The syntax here is circular and confusing. Please reword for clarity.

• Line 198: "12 and the Cochran's Q test." This is a typo for I2 (heterogeneity index). Please correct it to I2.

• Line 265-266: "by 3 %... in studies with sample size comprised between 100 and 499". There is a stray space in "3 %", and the formatting should be tightened.

• Line 268: Missing a period at the end of the sentence ("...sample size ≧500).

• Line 314: "The infection rates gradually declined over nearly 30 years..." However, the inclusion criteria strictly limited the search to papers published between 2000 and 2026 (a 26-year span). Modify the text to accurately reflect the timeline of your data.

• Table 3 formatting: The formatting of Table 3 is fragmented and messy, with recommendations awkwardly spanning cell boundaries or running across margins. It should be meticulously cleaned up to fit the journal’s stylistic requirements.

**********

what does this mean?). If published, this will include your full peer review and any attached files.

**Do you want your identity to be public for this peer review?**  If you choose “no”, your identity will remain anonymous but your review may still be made public.

For information about this choice, including consent withdrawal, please see our Privacy Policy

Reviewer #1: No

Reviewer #2: No

**********

[NOTE: If reviewer comments were submitted as an attachment file, they will be attached to this email and accessible via the submission site. Please log into your account, locate the manuscript record, and check for the action link "View Attachments". If this link does not appear, there are no attachment files.]

# Author comment: Author response to Decision Letter 1

**DOI:** 10.1371/journal.pgph.0006486.r002

9 Jun 2026

Attachment Submitted filename: Response to Reviewers_R1.docx

# Peer review: Decision Letter 1

**DOI:** 10.1371/journal.pgph.0006486.r003

**Authors:** Kiatichai Faksri

1 Jul 2026

PGPH-D-26-01598R1

Burden, clinical impact, and primary antimicrobial resistance profiles of Helicobacter pylori infections in Cameroon: a systematic review and meta-analysis

PLOS Global Public Health

Dear Dr. Kojom Foko,

Thank you for submitting your manuscript to PLOS Global Public Health. After careful consideration, we feel that it has merit but does not fully meet PLOS Global Public Health’s publication criteria as it currently stands. Therefore, we invite you to submit a revised version of the manuscript that addresses the points raised during the review process.

Please submit your revised manuscript by Jul 31 2026 11:59PM. If you will need more time than this to complete your revisions, please reply to this message or contact the journal office at globalpubhealth@plos.org. When you're ready to submit your revision, log on to https://www.editorialmanager.com/pgph/ and select the 'Submissions Needing Revision' folder to locate your manuscript file.

Please include the following items when submitting your revised manuscript:

- A letter that responds to each point raised by the editor and reviewer(s). You should upload this letter as a separate file labeled 'Response to Reviewers'.
- A marked-up copy of your manuscript that highlights changes made to the original version. You should upload this as a separate file labeled 'Revised Manuscript with Track Changes'.
- An unmarked version of your revised paper without tracked changes. You should upload this as a separate file labeled 'Manuscript'.

Guidelines for resubmitting your figure files are available below the reviewer comments at the end of this letter.

As the corresponding author, your ORCID iD is verified in the submission system and will appear in the published article. PLOS supports the use of ORCID, and we encourage all coauthors to register for an ORCID iD and use it as well. Please encourage your coauthors to verify their ORCID iD within the submission system before final acceptance, as unverified ORCID iDs will not appear in the published article. *Only*  the individual author can complete the verification step; PLOS staff *cannot*  verify ORCID iDs on behalf of authors.

We look forward to receiving your revised manuscript.

Kind regards,

Kiatichai Faksri, Ph.D

Academic Editor

PLOS Global Public Health

Journal Requirements:

If the reviewer comments include a recommendation to cite specific previously published works, please review and evaluate these publications to determine whether they are relevant and should be cited. There is no requirement to cite these works unless the editor has indicated otherwise.

Please review your reference list to ensure that it is complete and correct. If you have cited papers that have been retracted, please include the rationale for doing so in the manuscript text, or remove these references and replace them with relevant current references. Any changes to the reference list should be mentioned in the rebuttal letter that accompanies your revised manuscript. If you need to cite a retracted article, indicate the article’s retracted status in the References list and also include a citation and full reference for the retraction notice.

Additional Editor Comments (if provided):

[Note: HTML markup is below. Please do not edit.]

Reviewers' comments:

Reviewer's Responses to Questions

**Comments to the Author**

Reviewer #1: All comments have been addressed

Reviewer #2: All comments have been addressed

**********

publication criteria?

Reviewer #1: Yes

Reviewer #2: Yes

**********

3. Has the statistical analysis been performed appropriately and rigorously?

Reviewer #1: I don't know

Reviewer #2: Yes

**********

4. Have the authors made all data underlying the findings in their manuscript fully available (please refer to the Data Availability Statement at the start of the manuscript PDF file)?

The PLOS Data policy

Reviewer #1: Yes

Reviewer #2: Yes

**********

5. Is the manuscript presented in an intelligible fashion and written in standard English?

Reviewer #1: Yes

Reviewer #2: Yes

**********

Reviewer #1: I confirm that the authors have addressed and satisfactorily completed all the revisions requested in my previous review. All comments and suggestions have been carefully considered, and the revised manuscript reflects the relevant changes. I have no further objections and recommend acceptance of the manuscript

Reviewer #2: Authors had been successfully improved several critical points from the initial submission.

Major

1.Although the authors had been discuss Amoxicillin Resistance in detail, the pooled resistance rate of 93.4% remains extremely difficult to reconcile with global literature. The authors should further clarify which susceptibility testing standards were used (EUCAST, CLSI, others),whether disc diffusion methods were employed,How resistance definitions differed across studies? Whether sensitivity analyses excluding older studies or lower-quality studies change the estimate.

2.The pooled prevalence analysis reports: I² ≈ 97%.This level of heterogeneity is exceptionally high. Although meta-regression was conducted, much unexplained heterogeneity remains. The Discussion should better emphasize that: (1)Pooled estimates must be interpreted cautiously,(2)the summary prevalence may not represent the national burden accurately, regional and methodological differences likely contribute substantially.

3.Four regions of Cameroon remain unrepresented. Most evidence comes from Centre and Littoral, limited National Representativeness. The authors should explicitly state that the pooled estimate primarily reflects populations from a limited geographic area rather than the entire country.

4.The conclusions should emphasize that these observations including H. pylori infection is associated with anemia, elevated cholesterol , elevated LDL are preliminary and hypothesis-generating rather than definitive due to evidence comes from only a few studies,

Minor

1. Language Editing

Numerous grammatical issues persist, for example:

"progresses" → "advances"

"analyzes" → "analyses"

Several awkward sentences throughout Introduction and Discussion.

Professional English editing is still recommended.

2. Reference Verification

Several references are very recent (2025–2026).

The journal should verify:

accuracy,DOI validity,publication status.

3. Statistical Reporting

Some p-values are listed as:p = 0

These should be reported as:p < 0.001

**********

what does this mean?). If published, this will include your full peer review and any attached files.

**Do you want your identity to be public for this peer review?**  If you choose “no”, your identity will remain anonymous but your review may still be made public.

For information about this choice, including consent withdrawal, please see our Privacy Policy

Reviewer #1: No

Reviewer #2: **Yes:**  Yu-Jen Fang

**********

[NOTE: If reviewer comments were submitted as an attachment file, they will be attached to this email and accessible via the submission site. Please log into your account, locate the manuscript record, and check for the action link "View Attachments". If this link does not appear, there are no attachment files.]

# Author comment: Author response to Decision Letter 2

**DOI:** 10.1371/journal.pgph.0006486.r004

9 Jul 2026

Attachment Submitted filename: Response to Reviewers_R2.docx

# Peer review: Decision Letter 2

**DOI:** 10.1371/journal.pgph.0006486.r005

**Authors:** Kiatichai Faksri

27 Jul 2026

Burden, clinical impact, and primary antimicrobial resistance profiles of Helicobacter pylori infections in Cameroon: a systematic review and meta-analysis

PGPH-D-26-01598R2

Dear Dr Kojom Foko,

We are pleased to inform you that your manuscript 'Burden, clinical impact, and primary antimicrobial resistance profiles of Helicobacter pylori infections in Cameroon: a systematic review and meta-analysis' has been provisionally accepted for publication in PLOS Global Public Health.

Before your manuscript can be formally accepted you will need to complete some formatting changes, which you will receive in a follow up email. A member of our team will be in touch with a set of requests.

Please note that your manuscript will not be scheduled for publication until you have made the required changes, so a swift response is appreciated.

IMPORTANT: The editorial review process is now complete. PLOS will only permit corrections to spelling, formatting or significant scientific errors from this point onwards. Requests for major changes, or any which affect the scientific understanding of your work, will cause delays to the publication date of your manuscript.

If your institution or institutions have a press office, please notify them about your upcoming paper to help maximize its impact. If they'll be preparing press materials, please inform our press team as soon as possible -- no later than 48 hours after receiving the formal acceptance. Your manuscript will remain under strict press embargo until 2 pm Eastern Time on the date of publication. For more information, please contact globalpubhealth@plos.org.

Thank you again for supporting Open Access publishing; we are looking forward to publishing your work in PLOS Global Public Health.

Best regards,

Kiatichai Faksri, Ph.D

Academic Editor

PLOS Global Public Health

***********************************************************

Reviewer Comments (if any, and for reference):

Reviewer's Responses to Questions

**Comments to the Author**

Reviewer #2: All comments have been addressed

**********

publication criteria?

Reviewer #2: Yes

**********

3. Has the statistical analysis been performed appropriately and rigorously?

Reviewer #2: Yes

**********

4. Have the authors made all data underlying the findings in their manuscript fully available (please refer to the Data Availability Statement at the start of the manuscript PDF file)?

The PLOS Data policy

Reviewer #2: Yes

**********

5. Is the manuscript presented in an intelligible fashion and written in standard English?

Reviewer #2: Yes

**********

**Reviewer #2:**  The authors have meticulously revised the manuscript (R2) and addressed all major methodological and interpretive concerns raised during previous review rounds.

The addition of sub-analyses for antimicrobial resistance, detailed discussions regarding high heterogeneity (I^2 = 97.1%), and clear boundaries regarding the geographic generalizability of the findings have significantly strengthened the paper's rigor.

Minor Points for Final Polishing:

Clinical Actionability vs. Methodology (Amoxicillin Resistance):

Given that amoxicillin remains the backbone of primary eradication regimens (e.g., dual or quadruple therapies), please ensure the text clearly highlights that the unusually high amoxicillin resistance (93.4%) is likely a methodological artifact of disc diffusion and should not lead clinicians to discard amoxicillin-based therapy without MIC or molecular confirmation.

Grammar & Formatting Checklist:

Line 66: Change "progresses" to "advances". Line 161 / Line 180: Change "analyzes" to "analyses". Standardize the decimal places for reported $p$-values across tables and text (e.g., p = 0.43 instead of p = 0.4257).

**********

what does this mean?). If published, this will include your full peer review and any attached files.

**Do you want your identity to be public for this peer review?**  If you choose “no”, your identity will remain anonymous but your review may still be made public.

For information about this choice, including consent withdrawal, please see our Privacy Policy

Reviewer #2: **Yes:**  None

**********
